# Influence of edible flower inclusion on the nutritional and flavor changes of fermented pear wine

**DOI:** 10.3389/fnut.2025.1604754

**Published:** 2025-07-21

**Authors:** Mingjing Yang, Jintao Wang, Li Li, Qirui Xiong, Xiaofei Li, Xuhong Zhou

**Affiliations:** Office of Science and Technology, Yunnan University of Chinese Medicine, Kunming, China

**Keywords:** pear, rose, wine, phenolic compounds, flavonoids, esters antioxidant activity, tyrosinase inhibition

## Abstract

Compound-fermented wines integrate the aromas, flavor compounds, and nutritional components from several raw materials, enriching the flavor and texture of the final product. This study aimed to explore the influence of edible flowers on the quality of pear wine by evaluating the total phenol and flavonoid contents, antioxidant capacities, and tyrosinase inhibition abilities during the mixed fermentation of pears (P) with *Rosa rugosa* (PR), *Dendrobium candidum* (PD), *Chrysanthemum morifolium* (PC), *Lonicera japonica* (PL), and *Osmanthus fragrans* Lour (PO), using standard methods The findings revealed that total phenol and flavonoid contents, antioxidant capacities, and tyrosinase inhibition abilities significantly increased in compound pear-flower wine. Specifically, total polyphenol content (TPC), total flavonoid content (TFC), the DPPH(1,1-diphenyl-2-picryl-hydrazyl radical) radical scavenging activity (DRSA), Trolox equivalent antioxidant capacity (TEAC), and ferric reducing antioxidant power (FRAP) for PR were 580.69 ± 9.51 mg of gallic acid equivalents (GAE) per liter of the sample (mg GAE/L), 600.05 ± 36.6 mg of rutin per liter of the sample (mg RE/L), 0.51 ± 0.00 μmol of Trolox equivalents (TE) per milliliter (μmol TE/mL), 10.11 ± 0.06 μmol TE/mL and 6.35 ± 0.35 μmol of Fe^2+^ equivalents (FE) per milliliter (μmol FE/mL), respectively. Additionally, we further analyzed the volatile and non-volatile components of P and PR at different fermentation stages. A significant difference was observed between the non-volatile and volatile metabolites, with pear rose wine (PRW) demonstrating superior characteristics compared with pear wine (PW). Phenolic acids and flavonoids were closely associated with the formation of non-volatile metabolites, while esters, hydrocarbons, alcohols, and ketones were significantly linked to volatile formation. Notably, 2(5H)-furanone, 5-ethyl-3-hydroxy-4-methyl-, emerged as a significant aroma contributor with a relative odor activity value (ROAV) of 236,348.11, giving a sweet, fruity, nutty taste. Compared with PR, decanoic acid ethyl ester increased 634.67-fold in PRW. These findings provide a foundation for further exploration into optimized fermentation protocols, mechanistic studies on flavor and bioactive compound formation, and potential commercial applications in the functional beverage industry.

## 1 Introduction

Pear (*Pyrus communis* L.), belonging to the Rosaceae family and Pomoideae subfamily, is a plant with both culinary and medicinal applications (1). It is one of the three main fruits in China, with its cultivation area, production, export volume, and variety diversity ranking among the top in the world (2). Pears have been used as herbal medicine for more than 2000 years, valued for their ability to soothe coughs, support lung health, promote bowel regularity, and mitigate the effects of alcohol consumption (1, 3). Edible flowers are considered non-toxic and safe for human consumption and have been associated with nutritional, medicinal, and cosmetic benefits (4, 5). Some reported edible flowers, such as species of *Rosa, Chrysanthemum, Dendrobium*, and *Osmanthus*, have been shown to be beneficial to human health, offering properties such as antioxidant, anti-cancer, and anti-inflammatory effects (5, 6). The beneficial health effects of edible flowers are closely related to the presence of phytochemicals such as phenolic acids, flavonoids (including anthocyanins), alkaloids, phenylpropanoids, and terpenoids, which have multiple pharmacological properties (6).

Fruit wine fermentation is a biochemical process in which yeasts metabolize sugars, converting them into alcohol, esters, and other secondary metabolites (7). However, traditional fruit wine is usually made by fermenting a single type of fruit, resulting in a homogeneous taste and limited nutritional value (8). In contrast, compound fruit wine is prepared by fermenting a mixture of different fruits or by combining fruits with botanicals, which enhances the nutritional content and adds complexity to the flavor profile. This makes compound fruit wine highly competitive in the wine market due to its high quality and rich taste (9). Polyphenols play a significant role in both the non-volatile and volatile composition of fruit wine, contributing considerably to its color, mouthfeel, aroma, and flavor (10). Metabolites, which form the foundation of an organism's phenotype, are crucial for understanding biological processes and mechanisms more intuitively and effectively (11). Metabolomics, which involves the qualitative and quantitative analysis of metabolites, is used to study metabolic pathways or networks, investigate the metabolism of different biological phenotypes, understand the response mechanisms of metabolites to physical, chemical, or pathogenic stimuli, and evaluate food and drug safety (12). Recent studies have utilized extensive targeted metabolomics and multivariate statistical analysis to identify and analyze non-volatile metabolites in different compound wines. For example, Wang et al. studied the types and concentrations of non-volatile metabolites in *Lycium barbarum* and *Polygonatum cyrtonema* compound wine (13). Liu et al. analyzed the metabolites and antioxidant activities in lycopene-enriched compound fruit wine (14). However, research on pear-based fruit wine combined with edible flowers is still limited. While previous studies on pear wine focused on single fermentation and process optimization (15), our work pioneers the co-fermentation of pears or edible flowers, demonstrating significant improvements in aromatic complexity and polyphenol retention. Developing a new type of fruit wine that combines the flavors and nutritional benefits of pears and edible flowers could meet consumer demand for nutritional and functional products. Nevertheless, the effect of edible flowers on the quality of fruit wine remains unclear.

This study aimed to investigate the changes in non-volatile and volatile metabolites during the fermentation process using an ultra-performance liquid chromatography–tandem mass spectrometry (UPLC-MS/MS)-based widely targeted metabolomic approach combined with gas chromatography–tandem mass spectrometry (GC-MS/MS). The orthogonal partial least squares discriminant analysis (OPLS-DA) model was employed to identify significant differences in non-volatile and volatile metabolites before and after fermentation, elucidating the effects of fermentation on the quality, flavor, and antioxidant activities of PW. The findings will contribute to stabilizing the quality of flower–fruit wine, enhancing the understanding of flavor formation mechanisms, and guiding the standardized production of flower–fruit wines.

## 2 Materials and methods

### 2.1 Experimental materials

Fresh *Rosa rugosa, Dendrobium candidum, Chrysanthemum morifolium, Lonicera japonica*, and *Osmanthus fragrans* Lour flowers were obtained from Kunming, Yunnan, China (102°2′E; 25°2′N) and dried at 45°C in a drying oven (GZX-9240MBE). The flowers grow in red soil, and during their growth, compound fertilizers containing nitrogen, phosphorus, and potassium are applied. In the vegetative growth stage, insect repellents are used when insects are present. However, once the flowers bloom, the insect repellent is no longer used. Fresh, ripe, and pest-free pears were also obtained from Kunming, Yunnan, China. The pears grow in red soil, and during their growth, compound fertilizers containing nitrogen, phosphorus, and potassium are applied. In the vegetative growth stage, insect repellents are used when insects are present. However, once the pears start to bear fruit, the insect repellent is no longer used. The commercial yeast used for making compound fruit wine was *Saccharomyces cerevisiae* (Angel RW type, Angel Yeast Co., Ltd., Yichang, China).

### 2.2 Preparation of the compound pear–flower wine

The preparation of the flower–pear wine was assessed using a previously reported method with slight modifications (16, 17). The pears were washed to remove dirt and dust, then cored, peeled, and chopped. The chopped pears were mixed separately with *Rosa rugosa, Dendrobium candidum, Chrysanthemum morifolium, Lonicera japonica*, and *Osmanthus fragrans* Lour at a ratio of 400:1 (w/w). Each mixture was then juiced using an electric juicer (SUPOR, SP503A). To inhibit the growth of undesirable microorganisms and prevent browning, 50 mg/L of potassium metabisulphite (Macklin, China) was added to each juice. Lallzyme EX-V pectinase (20 mg/L, Scott Laboratories, Petaluma, California, USA) was added to accelerate enzymatic hydrolysis, prevent gelation, and increase juice yield, and the mixture was maintained at 40°C for 2 h (17). The initial sugar content in the fruit was 12°Brix, which was adjusted to 20 °Brix by adding sucrose. Before fermentation, dry yeast (0.2 g/L) was activated in a 5% glucose solution at 37°C for 30 min with continuous stirring. The activated yeast was then thoroughly mixed with the juice samples, and sealed fermentation was carried out at 26°C. Samples were collected after juicing (recorded as D0) and then aseptically taken on days 2, 4, 6, 8, and 10 for analysis. The collected samples were stored at −80°C until further analysis. Based on the content of polyphenols and flavonoids, as well as *in vitro* antioxidant and tyrosinase activities produced during fermentation, the best edible flower-fruit wine was selected for further analysis of non-volatile and volatile metabolites.

### 2.3 Determination of pH value and soluble solids content

The pH of the samples was measured using a pH meter (Hanna Instruments, Ann Arbor, Michigan, USA) by inserting the electrode directly into the sample solutions at ambient temperature. The soluble solids content was determined using a handheld refractometer, with readings calibrated to a temperature of 20°C.

### 2.4 Total polyphenol and total flavonoid contents

The TPC was measured using the Folin–Ciocalteu colorimetric method following a previously described protocol (18). Briefly, an appropriate amount of the sample was placed in a clean centrifugal tube, then 0.5 mL of Folin–Ciocalteu reagent was added and mixed. Finally, 1 mL of 7.5% Na_2_CO_3_ solution was added, and the volume was adjusted to 10 mL with distilled water. After 35 min of incubation in the dark, the mixture was centrifuged at 8,000 rpm at 4°C for 10 min. The absorbance was determined at 765 nm. The TPC was expressed as mg GAE/L.

The TFC was determined based on a previously reported method with slight modifications (19). In brief, 0.5 mL of the test samples was placed in a 10-mL centrifuge tube. Then 0.5 mL of NaNO_2_ solution (5%) was added, mixed, and held at room temperature for 5 min. Approximately 0.5 mL of AlCl_3_ solution (10%) was added, and the mixture was incubated for 6 min. Next, 5 mL of NaOH (1 M) was added, and the volume was adjusted to 10 mL with water. The solution was vortexed thoroughly and incubated for 10 min, after which it was centrifuged at 8,000 rpm at 4°C for 10 min. The absorbance was then measured at 510 nm. Rutin was used to create a standard curve, and TFC was reported as mg RE/L.

### 2.5 Determination of *in vitro* antioxidant activity

The DRSA of the fermented sample was evaluated according to a previously reported method. In brief, 2 mL of 79 μmol/L DPPH–methanol solution was mixed with 0.5 mL of the fermented liquor. The mixture was then incubated in the dark for 10 min at ambient temperature, and the absorbance was read at 517 nm, using a standard curve prepared with Trolox. The results were expressed as μmol TE/mL.

TEAC and FRAP were based on previously reported procedures with slight modifications (20, 21). In brief, the reaction solution contained 100 μL of fermentation liquor and 3.8 mL of ABTS working solution. The TEAC absorbance was recorded at 734 nm, with values expressed as μmol TE/mL based on a Trolox standard curve. For the FRAP assay, the reaction solution contained 100 μL of samples and 3 mL of FRAP working solution. It was incubated at 37°C for 4 min, and the absorbance was measured at 593 nm, using ferrous sulfate as a reference standard. The results were expressed as μmol FE/mL.

### 2.6 Tyrosinase inhibition activity

Tyrosinase (TYR) inhibition activity was assessed using a previously reported method with slight modifications (22). Briefly, 75 μL of the sample was mixed with 25 μL of tyrosinase solution (1 mg/mL) and incubated at 37°C for 10 min. Following incubation, 100 uL of 1 mol/L L-DOPA solution was added to initiate the reaction at 37°C for 5 min, and the absorbance was measured at 475 nm. Kojic acid was used as a positive control. TYR inhibition activity was calculated using Equation 1:


(1) $$\begin{array}{l} \%inbition=\left[\left(Ab-A\right)-\left(Cb-C\right)\right]/\left(Cb-C\right)\times 100 \end{array}$$

where Ab is the absorbance of the sample solution with the tyrosinase solution, A2 is the absorbance of the sample solution with sodium phosphate buffer (pH 6.8), Cb is the absorbance of the sodium phosphate buffer (pH 6.8) with the tyrosinase solution, and C is the absorbance of the sodium phosphate buffer alone (pH 6.8).

### 2.7 Non-volatile metabolite analysis

#### 2.7.1 Ultra performance liquid chromatography conditions

Based on the indicators of total phenol and flavonoid content, antioxidant capacity, and tyrosinase inhibition ability of the comprehensive compound wine, it was found that the P mixed with Rose (PR) showed the highest values among the sample groups. The samples analyzed included unfermented pear juice (UP) and unfermented rose pear juice (UPR), as well as fermented samples collected on days 4 (FP4 and FPR4), days 6 (FP6 and FPR6), and days 8 (FP8 and FPR8) for PW and PRW, respectively. The sample extracts were analyzed using a UPLC-ESI-MS/MS system (UPLC, ExionLC™ AD, https://sciex.com.cn/) and tandem mass spectrometry system (https://sciex.com.cn/). The analytical conditions were as follows: UPLC column, Agilent SB-C18 (1.8 μm, 2.1 mm ^*^ 100 mm); the mobile phase consisted of solvent A, pure water with 0.1% formic acid, and solvent B, acetonitrile with 0.1% formic acid. Sample measurements were performed with a gradient program that employed the starting conditions of 95% A and 5% B. Within 9 min, a linear gradient to 5% A and 95% B was programmed, and a composition of 5% A and 95% B was kept for 1 min. Subsequently, a composition of 95% A and 5.0% B was adjusted within 1.1 min and kept for 2.9 min. The flow rate was set to 0.35 mL per minute; the column oven was set to 40°C; and the injection volume was 2 μL. The effluent was alternatively connected to an ESI-triple quadrupole-linear ion trap (QTRAP)-MS.

#### 2.7.2 Electrospray ionization–triple quadrupole linear ion trap mass spectrometer

The ESI source operation parameters were as follows: source temperature 500°C; ion spray voltage (IS) 5,500 V (positive ion mode)/-4,500 V (negative ion mode); ion source gas I (GSI), gas II (GSII), and curtain gas (CUR) were set at 50, 60, and 25 psi, respectively; the collision-activated dissociation (CAD) was set to high. QQQ scans were acquired as MRM experiments with collision gas (nitrogen) set to medium. DP (declustering potential) and CE (collision energy) for individual MRM transitions were optimized with further DP and CE adjustments. A specific set of MRM transitions was monitored for each period according to the metabolites eluted during this time.

### 2.8 Volatile metabolite analysis

#### 2.8.1 Solid-phase microextraction–gas chromatography–tandem mass spectrometry

The sample preparation for volatile metabolite analysis was conducted following the procedure described in a previous study with slight modifications (23). 1 mL of the sample was transferred immediately to a 20 mL head-space vial (Agilent, Palo Alto, CA, USA) containing a NaCl saturated solution to inhibit any enzymatic reactions. The vials were sealed using crimp-top caps with TFE-silicone headspace septa (Agilent). At the time of SPME analysis, each vial was placed at 60°C for 5 min, then a 120 μm DVB/CWR/PDMS fiber (Agilent) was exposed to the headspace of the sample for 15 min at 60°C. A 7890B-7000C system (Agilent, CA, USA) equipped with a DB-5MS ultra-inert capillary column (30 m × 0.25 mm × 0.25 μm) was used. High-purity helium (99.999%) served as the carrier gas at a constant flow rate of 1.2 mL/min. The temperature of the gas chromatography (GC) injector was set at 250°C. The following column temperature program was employed: the initial temperature was set at 40°C for 3.5 min, then increased to 100°C at a rate of 10°C/min and held for 5 min, followed by an increase to 180°C at a rate of 7°C/min, and finally increased to 280°C at a rate of 25°C/min and held for 5 min. Mass spectra were recorded in electron impact (EI) ionization mode at 70 eV. The quadrupole mass detector, ion source, and transfer line temperatures were set at 150, 230, and 280°C, respectively. The MS was operated in selected ion monitoring (SIM) mode for the identification and quantification of analytes.

#### 2.8.2 ROAV analysis

ROAV is a commonly used metric for evaluating the contribution of aroma compounds. ROAV values >1 are generally considered to indicate a significant contribution to the aroma profile of the sample. ROAV analysis was conducted following previously described procedures (24, 25). The ROAV was calculated using Equation 2:


(2) $$\begin{array}{l} ROAVi=\frac{Ci}{Ti} \end{array}$$

where ROAVi is the relative odor activity value of the compound, Ci is the relative content of the compound (μg/g or μg/mL), and Ti is the threshold of the compound in water (μg/g or μg/mL).

#### 2.8.3 Sensory analysis

The sensory evaluation of rose–pear compound wine was performed according to GB/T 15038-2006 *General Analytical Methods for Wine and Fruit Wine* (http://down.foodmate.net/standard/sort/3/11619.html). Ten trained sensory panelists scored the compound fruit wine based on four criteria: appearance, aroma, taste, and typicality, with a maximum score of 100 points. The detailed sensory scoring criteria are shown in Supplementary Table S13. Based on the differential metabolites identified according to screening criteria and the annotated sensory flavor characteristics in each comparison group, the top 10 sensory flavors with the highest frequency of annotations were selected for radar mapping, which was conducted following previously described procedures (26).

### 2.9 Statistical analysis

All results were presented as mean ± standard deviation (SD), with measurements conducted in independent experiments in triplicate. A *p*-value of *p* < 0.05 was considered statistically significant. Metabolomics multivariate statistical analysis was conducted using SIMCA software (version 14.1, Umetrics AB, Umeå, Västernorrland, Sweden).

## 3 Results and discussion

### 3.1 Evolution of soluble solids content and pH values during fermentation

**Figure 1:**
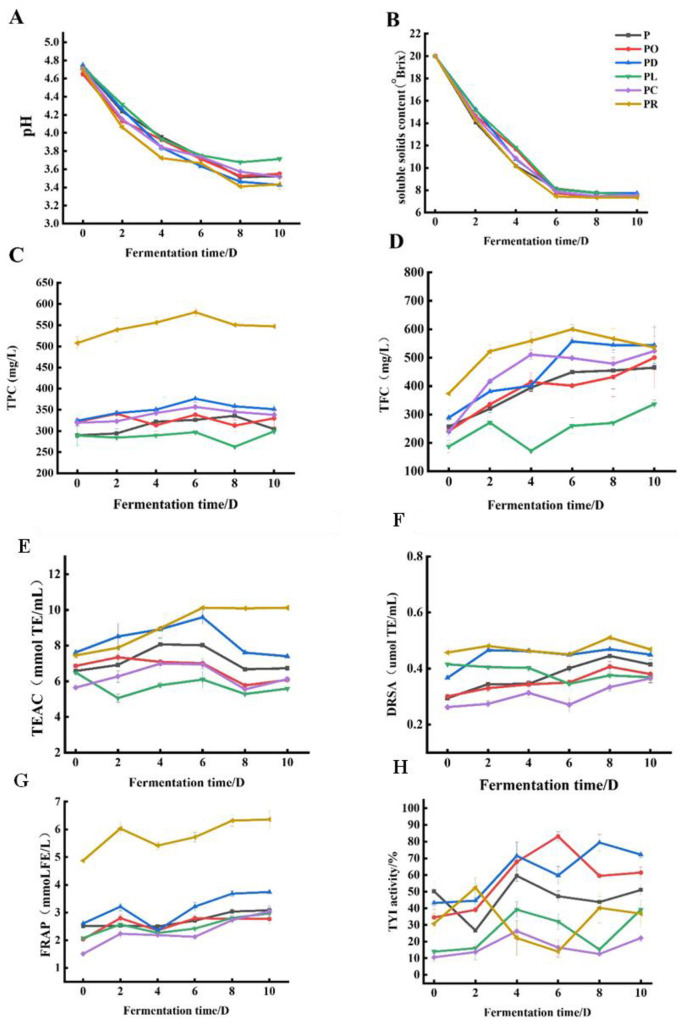
Change of flower–fruit wine during different fermentation stages. pH values **(A)**, soluble solids content **(B)**, TPC **(C)**, TFC **(D)**, the antioxidant abilities TEAC **(E)**, DRSA **(F)**, FRAP **(G)**, and TYR **(H)**.

The variation in pH values is crucial as it reflects the degree of fermentation (27). As shown in Figure 1A, the pH value of all samples decreased significantly in the early stage of fermentation and stabilized in the later stage, with PR (3.41 ± 0.01) being the lowest, likely due to the rapid growth and multiplication of yeasts in the favorable acidic environment. As fermentation continued, the pH values stabilized, showing minimal change due to the consumption of nutritional components (10). During fermentation from day 0 to day 6, the soluble solids content significantly decreased (*P* < 0.05). As shown in Figure 1B, the lowest recorded soluble solids content was 7.200 ± 0.0001 °Brix on day 6 for PR, representing a 64% decrease compared to day 0. In fruit wine fermentation, microorganisms convert sugars into ethanol and flavor compounds. For example, when making kiwi wine, additional sugar is often added before fermentation to increase the final alcohol content (24).

### 3.2 Total phenolic content and total flavonoid content

In recent years, microbial fermentation has emerged as a promising approach to enhance the release of bioactive compounds from plant sources (3, 56). The changes in TPC and TFC in the flower–fruit compound wine during fermentation are shown in Figures 1C, D. As fermentation progressed, TPC and TFC initially increased and then decreased. The initial increase in TPC and TFC may have resulted from the action of carbohydrate hydrolases produced by microorganisms, which promoted the release of insoluble and bound polyphenols during the early fermentation stage (27). As fermentation continued, the polyphenol content decreased after reaching a maximum value. This decrease could be due to the consumption of essential nutrients, such as carbon and nitrogen sources, from the edible flowers and fruits that supported the growth of microorganisms (28). Overall, the TPC and TFC of PR, PD, PO, PL, and PC increased after fermentation compared to their unfermented counterparts. Among these, PR had significantly higher TPC and TFC, while PL had the lowest values. Specifically, the maximum TPC and TFC values for PR were 580.69 ± 9.51 mg GAE/L and 600.05 ± 36.6 mg RE/L on the 6^th^ day of fermentation, accounting for 14.3% and 60.4% increases compared to the unfermented samples, respectively. The maximum TPC values, in descending order, were as follows: 376.29 ± 8.18 mg GAE/L (PD), 356 ± 5.56 mg GAE/L (PC), 338.40 ± 3.29 mg GAE/L (PO), 335 ± 15.75 mg GAE/L (P), and 299.14 ± 7.29 mg GAE/L (PL). Similarly, the maximum TFC values, in descending order, were: 557.08 ± 59.53 mg RE/L (PD), 551.83 ± 48.39 mg RE/L (P), 523.57 ± 37.40 mg RE/L (PC), 500.13 ± 106.84 mg RE/L (PO), and 336.98 ± 14.17 mg RE/L (PL). Enzymes produced by yeasts, such as pectinase and β*-*glycosidase, may influence the extraction of phenolic compounds from fruits and hydrolyze the glycosidic bonds in phenolic compounds, respectively (29). Phenolic compounds, including phenolic acids, flavonoids, amino acids and their derivatives, and anthocyanins, are primary determinants of fruit wine quality (30). TPC and TFC increased to varying degrees after fermentation, consistent with findings from studies on *Lycium barbarum* and *Polygonatum cyrtonema* compound wine (13).

### 3.3 Antioxidant abilities

The antioxidant activities of P, PO, PD, PL, PC, and PR were assessed using the DPPH, TEAC, and FRAP methods, as shown in Figures 1E–G. The antioxidant capacities generally followed a trend of initially increasing and then decreasing over the fermentation period. However, the FRAP assay showed a pattern of increase, followed by a decrease, and then another increase. This variation may be due to the different antioxidative attributes arising from different reaction mechanisms. The specificity and sensitivity of a single analytical method are insufficient to comprehensively detect all antioxidant constituents within the test sample. Therefore, it is suggested that these methods be used together with other methods to distinguish the dominant mechanisms for different antioxidants (31, 32). Notably, the antioxidant capacity of PR was significantly higher than that of P, PD, PO, PL, and PC. Specifically, the DRSA, TEAC, and FRAP results for PR reached 0.51 ± 0.00 μmol TE/mL on the 8^th^ day of fermentation, 10.11 ± 0.06 mmol TE/mL on the 6^th^ day, and 6.35 ± 0.35 mmol FE/mL on the 8^th^ day, respectively. These values accounted for 13.3%, 35.89%, and 30.40% of the corresponding values in the unfermented samples.

Phenolic compounds have been demonstrated to exhibit potent antioxidant activity. To rigorously elucidate the relationship between natural bioactive compounds and antioxidant capacity, comprehensive linear correlation analysis was carried out (33). To better understand the relationships among TPC, TFC, antioxidant capacity, and TYR inhibition activity, a correlation heatmap was generated, as shown in Supplementary Figure 1. The heatmap indicated a strong correlation between TPC and TFC with the different antioxidant capacities, suggesting that phenolics and flavonoids are the primary contributors to antioxidant activity. This correlation also explains the similar trends observed in the changes among TPC, TFC, and antioxidant capacities.

As shown in **Figure 4**, the metabolomics analysis shows that the main differential metabolites are classified as flavonoids and phenolic acids, with the majority demonstrating significant upregulated and exhibiting potent antioxidant properties, for instance, kaempferol-3-*O*-6′'-malonyl) glucoside, cinchonain Ib, vanillic acid methyl ester, gallic acid, and 2-hydroxycinnamic acid (34, 35).

### 3.4 Tyrosinase inhibition activity

Tyrosinase is a rate-limiting enzyme involved in melanin production, and inhibitors of this enzyme can regulate hyperpigmentation disorders by reducing melanin synthesis (22). As shown in Figure 1H, the tyrosinase inhibitory activity of the fermented samples (P, PO, PD, PL, PR, and PC) increased compared to their unfermented counterparts after yeast fermentation. Among these, PR showed the highest inhibitory activity, reaching 83.10 ± 2.84% on the 6^th^ day of fermentation, followed by PD, which reached its maximum (79.41 ± 4.95%) on the 8^th^ day. Extensive research has demonstrated that polyphenols and flavonoids, naturally occurring active compounds in plants, have potential tyrosinase inhibitory effects. Examples include kaempferol, cinnamic acid, isorhamnetin, quercetin, and morin, which can inhibit tyrosinase activity, while other compounds, such as catechin and rhamnetin, act as substrates that suppress tyrosinase activity, either by serving as cofactors (catechin) or by functioning as free radical scavengers (rhamnetin) (36). The findings of this study supported these observations. The correlation heatmap (Supplementary Figure 1B) showed a significant correlation between TFC, TPC, and tyrosinase inhibition activity in PR. Additionally, the correlation network diagram (Supplementary Figure 1G) showed that compounds such as kaempferol, quercetin, and cryptochlorogenic acid strongly correlated with TYR inhibition activity. These results indicated that phenolics and flavonoids may significantly contribute to the tyrosinase inhibitory effects observed in the fermented samples.

### 3.5 Non-volatile metabolites analysis

#### 3.5.1 Hierarchical clustering analysis and principal component analysis

**Figure 2:**
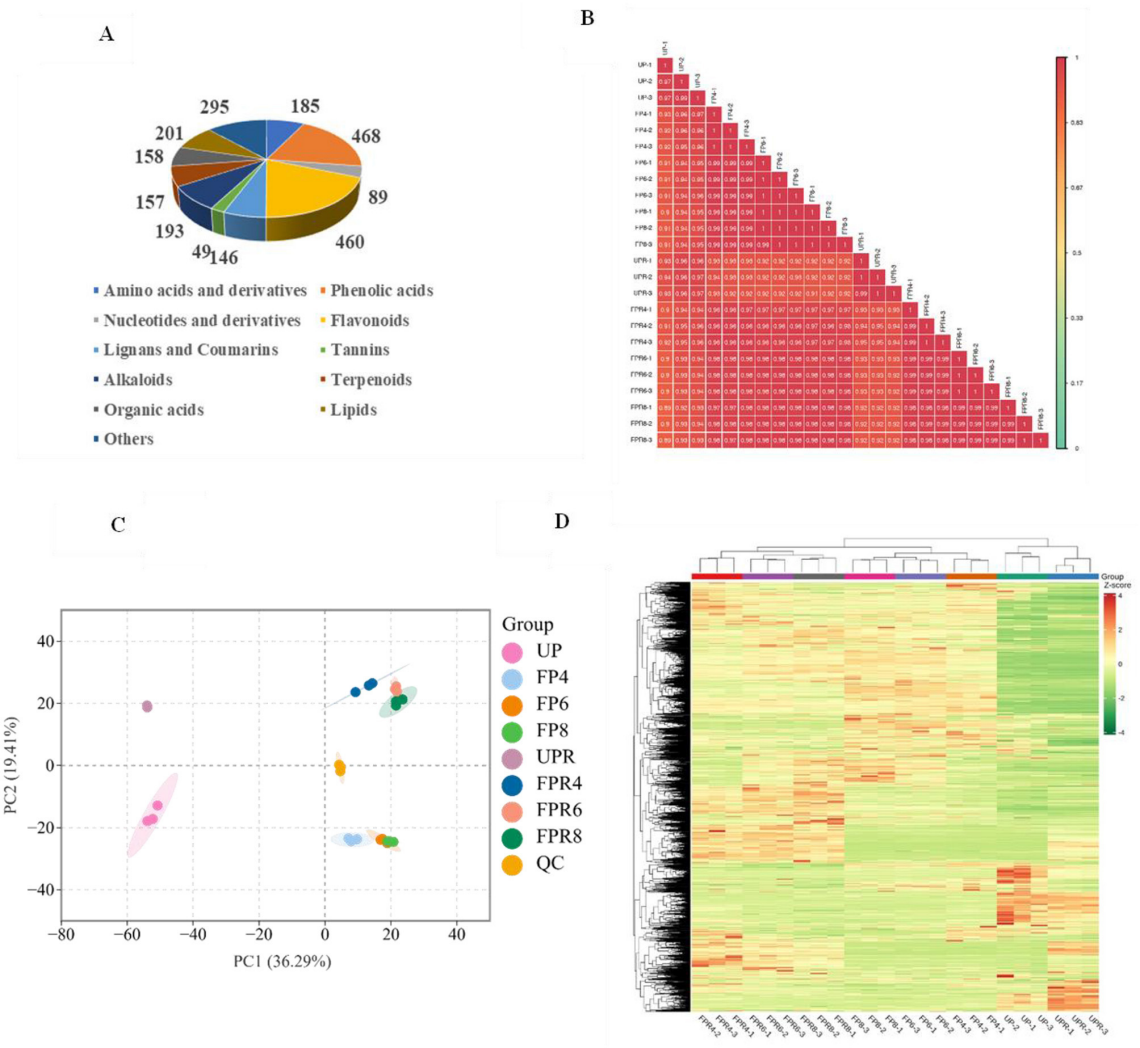
Classification of non-volatile metabolites **(A)**. Correlation analysis **(B)**. PCA score plot of all test samples **(C)**. Hierarchical cluster analysis (HCA) **(D)**.

Microbial fermentation can produce a variety of beneficial metabolites with biological activity. This study utilized a widely targeted metabolomic approach to investigate and identify differential metabolites as potential biomarkers in PW and PRW. Using the UPLC-MS/MS method, a total of 2,401 metabolites were identified, comprising 468 phenolic acids, 460 flavonoids, 193 alkaloids, 201 lipids, 185 amino acids and derivatives, 158 organic acids, 157 terpenoids, 146 lignans and coumarins, 89 nucleotides and derivatives, 49 tannins, and 295 other metabolites such as saccharides (Supplementary Table 1). Notably, 2,4-dihydroxybenzaldehyde was detected only in PW, while eudesmane-1β, 5α,11-triol, salirepin, methyl chebulagic acid, and 3,42′4′6′-pentahydroxychalcone4′-*O*-glucoside were found exclusively in PRW. Pie charts were generated to visualize the classification of these metabolites (Figure 2A).

A combination of PCA, HCA, and OPLS-DA was employed to investigate the intra- and inter-group differences in metabolite profiles and to improve the interpretability and reliability of the data. Correlation analysis of the secondary metabolites among different samples revealed a high positive correlation (Figure 2B). As shown in Figure 2C, the unfermented samples (UP and UPR) were distinguished from the fermented samples (FP and FPR), indicating significant differences in non-volatile metabolites before and after fermentation. Meanwhile, the PW samples were located below the QC value, while the PRW samples were above the QC value, indicating significant differences in the non-volatile metabolite profiles between PW and PRW. HCA further supported these findings, with independent clusters formed for the fermented and unfermented groups, demonstrating significant differences in metabolic profiles before and after fermentation. Additionally, two distinct clusters were observed for PW and PRW, indicating significant differences in metabolic profiles between these sample groups. Interestingly, clusters were observed between UP and UPR, which may be attributed to their shared origin from pear derivatives (Figure 2D). Overall, the HCA clustering and PCA analysis confirmed the reliability of the experimental data and revealed biologically significant differences in the metabolite profiles across different samples, effectively distinguishing between them, similar to the findings from previous studies (13). This finding provides a foundation for the subsequent screening of differential metabolites.

#### 3.5.2 Dynamic changes in non-volatile metabolites during fermentation

Partial least squares discrimination analysis (PLS-DA) was performed to better understand the dynamic changes in each class of non-volatile metabolites during fermentation. The fermented samples of PW were located on the left side of the plot, with FP6 and FP8 positioned in the upper left and FP4 in the lower left, indicating clear separations between different fermentation groups and between fermented and unfermented samples. In comparison, the difference between FP6 and FP8 was minimal, suggesting that day 4 of fermentation is the most critical point in the transformation of non-volatile metabolites in PW (Supplementary Figure 2A). Similarly, for PRW, a clear separation was observed between fermented and unfermented samples, as well as among the different fermentation groups (Supplementary Figure 2B). In this study, 2,401 substances were identified and classified into major differential metabolite groups in PW and PRW, including phenolic acids, flavonoids, amino acids and derivatives, lipids, alkaloids, lignans and coumarins, nucleotides and derivatives, organic acids, tannins, terpenoids, and other compounds. Changes in the levels of these 11 classes of non-volatile metabolites were monitored during fermentation. The total amounts of non-volatile metabolites exhibited different trends between PW and PRW (Supplementary Table 2). In PW, the levels of these metabolites showed a pattern of increase, followed by a decrease, and then a subsequent increase (Supplementary Figure 2C). In contrast, PRW showed an initial increase, followed by a subsequent decrease (Supplementary Figure 2D). These findings indicated that the metabolic profiles of non-volatile metabolites varied significantly before and after fermentation. The observed changes may be attributed to various enzymes secreted by yeasts, which can promote the metabolic progression of the entire fermentation system (10). In our study, phenolic acids and flavonoids were found to be the most abundant secondary metabolites in both PW and PRW, consistent with previous findings in the fermentation of roselle wine (16). Phenolics participate in several reactions during winemaking, such as cycloaddition, polymerization, and oxidation, leading to the formation of new compounds like ellagitannins, ethyl-bridged anthocyanin–flavanol derivatives, and anthocyanins (37). Research has shown that phenolic compounds significantly impact the overall sensory quality of wine, including its flavor and texture (38).

#### 3.5.3 Comparative analysis of differential metabolites in different comparison groups

**Figure 3:**
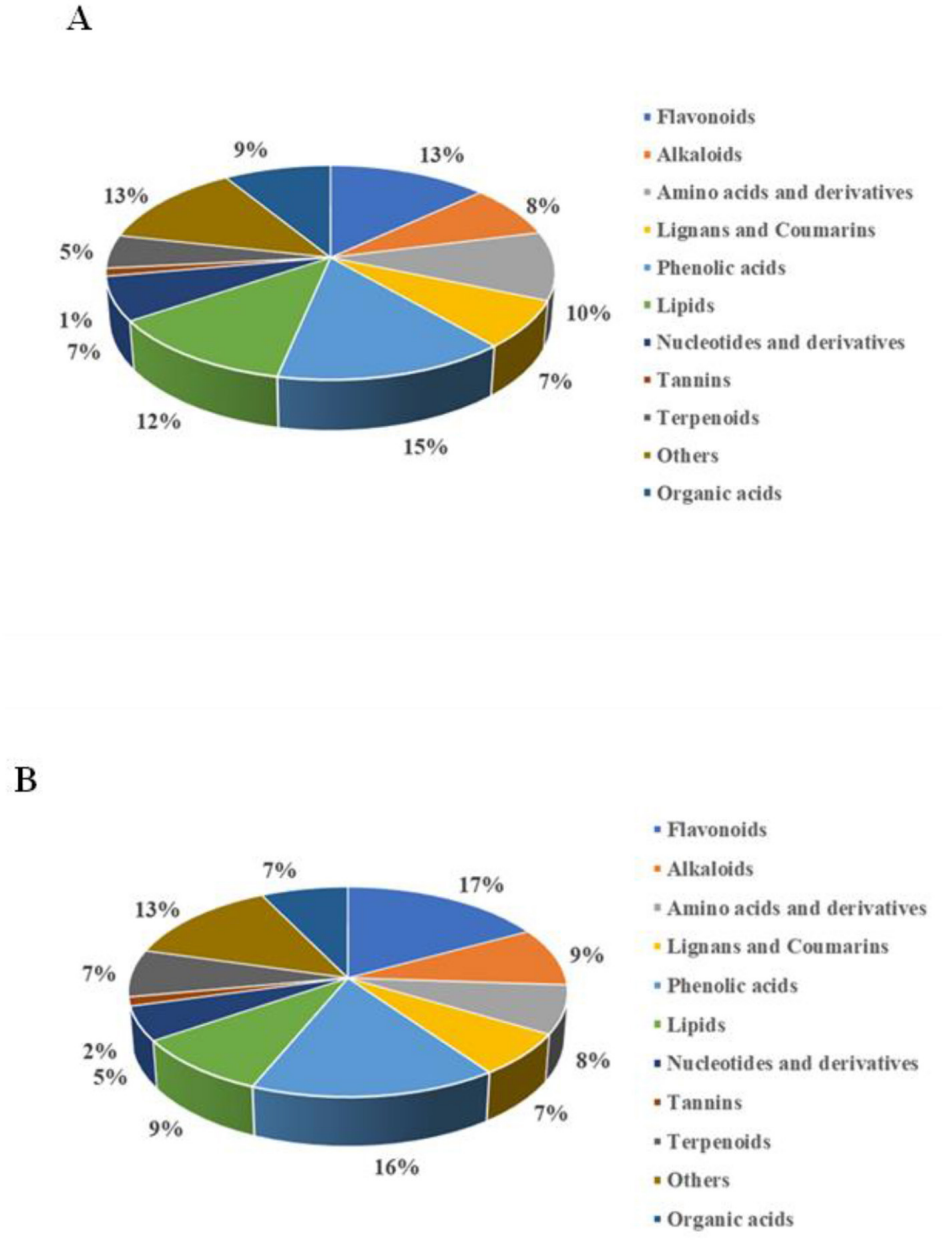
A comparison of significantly different non-volatile metabolites of PW **(A)** and PRW **(B)**.

A comparative analysis was conducted to identify the key differential metabolites between the fermentation groups. Compared to PCA and HCA, OPLS-DA offers supervised classification, which eliminates unrelated classification information and effectively monitors the transformation of metabolites over fermentation time (16). The variable importance in projection (VIP) value and fold change (FC) were used to identify differential metabolites based on OPLS-DA analysis (Supplementary Figure 3). Specifically, OPLS-DA was performed for both PW and PRW, using the criteria of VIP ≥ 1, FC >2 or FC < 0.5, and *p*-value ≤ 0.05. A total of 1,011 significantly different non-volatile metabolites were identified in PW, including 149 phenolic acids, 133 flavonoids, 124 lipids, 104 amino acids and derivatives, 89 organic acids, 80 alkaloids, 73 lignans and coumarins, 67 nucleotides and derivatives, 51 terpenoids, 12 tannins, and 129 other compounds (Figure 3A). The main differential metabolites in the phenolic acids group included dicaffeoylshikimic acid, 5-*O*-galloyl-methyl quinine ester, 2,3,4-trihydroxybutyl 6-*O*-(*E*)-caffeoyl-β-*D*-glucopyranoside, 3-*O*-feruloylquinic acid-*O*-glucoside, and glucosyloxybenzoic acid. In the flavonoids group, the main differential metabolites included diosmetin-7-*O*-galactoside, vitexin-2′'-*O*-glucoside, eriodictyol-3′-*O*-glucoside, and 1,2,4,5,8-pentahydroxy-6-methylanthracene-9,10-dione. For PRW, 1,155 significantly different non-volatile metabolites were identified, including 186 phenolic acids, 197 flavonoids, 106 lipids, 86 amino acids and derivatives, 84 organic acids, 104 alkaloids, 77 lignans and coumarins, 61 nucleotides and derivatives, 84 terpenoids, 16 tannins, and 154 other compounds (Figure 3B). The main differential metabolites in the phenolic acids group included methyl syringate, 5-*O*-β-*D*-glucopyranosyl-3-hydrobenzo(b)furan-2-one, digallic acid, 3-methoxybenzene-1,2-diol, and alnusonol. In the flavonoids group, the main differential metabolites included kaempferol-3-O-6′‘-malonyl glucoside,2′3′4′,5,7-pentahydroxyflavone, disporopsin, and cinchonain Ia. The differential metabolites between the two sample sets were visualized using volcano plots, revealing 911 significant differential metabolites for FP4 vs. UP, 227 for FP6 vs. FP4, 87 for FP8 vs. FP6, 1,171 for FPR4 vs. UPR, 210 for FPR6 vs. FPR4, and 123 for FP8 vs. FPR6 (Supplementary Figures 4-1A, B, 4-2A, B, Supplementary Tables S3, S8). These findings indicated that yeast fermentation is a crucial factor influencing nutrient interactions and the production of plant secondary metabolites in fruit wine (39). Additionally, the Venn diagrams showed the presence of both common and unique significant differential metabolites across the different fermentation groups. Specifically, FP4 vs. UP, FP6 vs. FP4, and FP8 vs. FP6 shared 47 common substances, while FPR4 vs. UPR, FPR6 vs. FPR4, and FPR8 vs. FPR6 shared 37 common substances (Supplementary Table S9, Supplementary Figures 4-1D, 4-2D). This indicates that phenolic acids and flavonoids play an important role in the flavor quality and health functions of fruit wine.

#### 3.5.4 Evolution of differential metabolites during fermentation of PW and PRW

**Figure 4:**
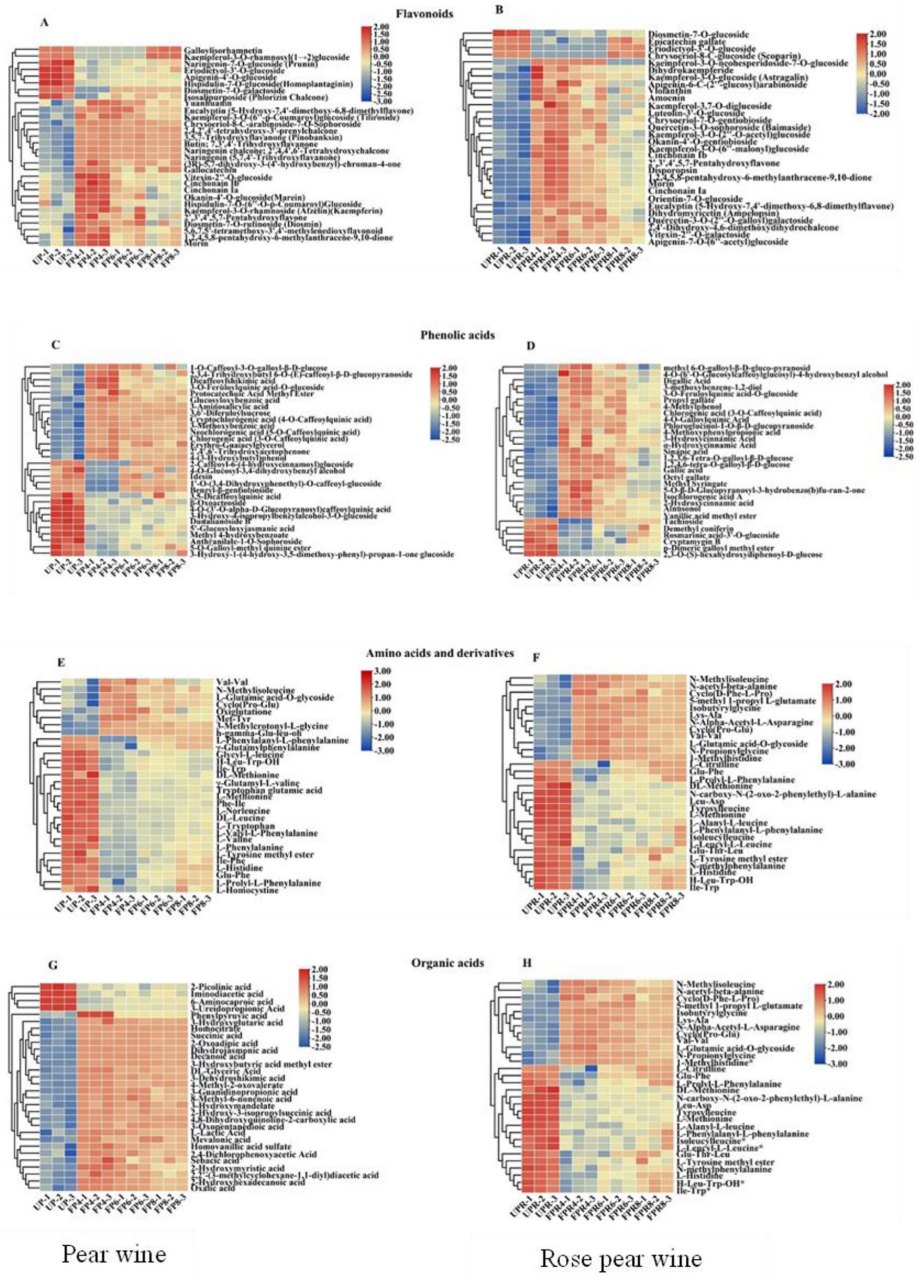
Heatmaps of the contents of significantly different flavonoid s **(A, B)**, phenolic acids **(C, D)**, amino acids and their derivatives **(E, F)**, organic acids **(G, H)** in PW and PRW during fermentation. Each colored cell corresponds to a value of different categories of non-volatile metabolites. Red color indicates high content, while green color indicates low content.

A heat map was generated to observe the significant changes in different metabolites (Figure 4), with the results presented below.

##### 3.5.4.1 Flavonoids

The major classes of flavonoids in wines are flavanols, flavonols, and anthocyanins (40, 41). Significant differences were observed among the 133 flavonoids identified in PW. Among them, the levels of vitexin2′‘-*O*-glucoside, cinchonain Ib, 1,2,4,5,8-pentahydroxy-6-methylanthracene-9,10-dione, and morin increased significantly after fermentation in PW, while diosmetin-7-*O*-galactoside and eriodictyol3′-*O*-glucoside showed a downward trend (Figure 4A). Significant differences were also observed among the 197 flavonoids in PRW. The flavonoids kaempferol-3-*O*-6′'-malonyl glucoside,2′3′4′,5,7-pentahydroxyflavone, cinchonain Ib, disporopsin, cinchonain Ia, 1,2,4,5,8-pentahydroxy-6-methylanthracene-9,10-dione, and morin showed significant increases after fermentation, while diosmetin-7-*O*-galactoside showed a downward trend during the fermentation process in PRW (Figure 4B). It is possible that the complex hydrolases produced by yeast during fermentation promoted the conversion of glycosylated anthocyanins and flavonols into their more biologically active aglycones (cyanidin and quercetin) (42).

##### 3.5.4.2 Phenolic acids

The impact of the polyphenol matrix on aroma is primarily realized through weak intermolecular non-covalent interactions, such as hydrogen bonding, dispersion forces, and hydrophobic effects, which promote the conversion of phenolic acids (43). Among the 149 phenolic acids examined in PW, dicaffeoylshikimic acid, 2,3,4-trihydroxybutyl 6-*O*-(E)-caffeoyl-β-*d*-glucopyranoside, 3-*O*-feruloylquinic acid-*O*-glucoside, and glucosyloxybenzoic acid significantly increased after FP4, while a downward trend was observed in FP6 and FP8. Additionally, 5-*O*-galloyl-methyl quinine ester and methyl 4-hydroxybenzoate showed a downward trend throughout the fermentation process (Figure 4C). In PRW, 186 phenolic acids were observed. Among them, methyl syringate, 5-*O*-β-D-glucopyranosyl-3-hydrobenzo(b)furan-2-one, digallic acid, 3-methoxybenzene-1,2-diol, alnusonol, 2-hydroxycinnamic acid, vanillic acid methyl ester, and gallic acid significantly increased after FPR4 and FPR6, while a downward trend was observed in FPR8. The p-dimeric galloyl methyl ester showed a downward trend throughout the fermentation process (Figure 4D). These results indicate that redox reactions, hydrolysis, and the activity of β-glucosidase, an important glycosidase produced by *Saccharomyces cerevisiae*, play crucial roles during fermentation, promoting the conversion of phenolic acids that significantly contribute to the color, astringency, taste, and overall flavor of fruit wine (39, 43).

##### 3.5.4.3 Amino acids and their derivatives

Amino acids, particularly aromatic amino acids, are among the primary classes detected in different fruit wines and serve as precursors for important volatile compounds in these wines (44). Among the 104 amino acids that showed significant changes in PW, L-histidine, glu-phe, L-valine, ile-phe, L-phenylalanine, L-phenylalanyl-L-phenylalanine, and L-homocystine showed a downward trend throughout the fermentation process. Conversely, met-tyr significantly increased after FP4, although a downward trend was observed in FP6 and FP8 (Figure 4E). In the PRW, 86 amino acids showed significant changes. L-histidine, h-leu-trp-oh, ile-trp, and glu-phe all showed a downward trend during fermentation. However, L-glutamic acid-*O*-glycoside and N-methylphenylalanine significantly increased after FPR4, with a downward trend observed in FPR6 and FPR8 (Figure 4F). Additionally, amino acids are considered vital nitrogen sources for the growth of *Saccharomyces cerevisiae* in the must. Their presence is a key factor in the browning of fruit wine through non-enzymatic mechanisms, such as the Maillard reactions (45). A deficiency in amino acids can decrease the operation of glucose permease, affecting sugar metabolism and reducing fermentation capacity. At the same time, yeast utilizes ketoacids produced during sugar metabolism to synthesize amino acids (46).

##### 3.5.4.4 Organic acids

Organic acids serve as key intermediate products of carbohydrate catabolism and are essential taste substances that significantly influence the sensory quality of fruit wine due to their sour taste and distinct flavor (23). During the fermentation process, α-ketoglutaric acid, 2-hydr, trans-2-butene-1,4-dicarboxylic acid, 6-hydroxyhexanoic acid, and mevalonic acid showed significant increases during fermentation (*p* < 0.05) (Figures 4G, H). Organic acids interact with other matrix components of fruit wine, such as ethanol, polyphenols, anthocyanins, and proteins, thereby impacting sensory attributes such as taste, color, and aroma. Moreover, the carboxyl group present in the organic acid structure becomes acidic upon dissociation, with its composition and concentration determining the pH value of the fruit wine and subsequently affecting its flavor.

#### 3.5.5 KEGG pathway annotation of differential metabolites

The pathways associated with various metabolites were analyzed using the KEGG database to investigate the mechanisms underlying metabolic changes in different samples. This study identified specific metabolic pathways related to the differential metabolites, and essential pathways were obtained using enrichment analysis. Compared to the UP group, the metabolic pathways in the FP4 group primarily included nucleotide metabolism, purine metabolism, glucosinolate biosynthesis, cofactor biosynthesis, tryptophan metabolism, and zeatin biosynthesis (Supplementary Figure 5A). In contrast, the FP6 group, when compared to FP4, demonstrated enhanced metabolism in aminoacyl-tRNA biosynthesis, isoquinoline alkaloid biosynthesis, phenylalanine metabolism, cyanoamino acid metabolism, galactose metabolism, and glucosinolate biosynthesis (Supplementary Figure 5B). Further comparison of FP8 with FP6 revealed a promotion of metabolic processes such as glutathione metabolism, flavone and flavonol biosynthesis, the biosynthesis of various alkaloids, ubiquinone and terpenoid–quinone biosynthesis, and cofactor biosynthesis (Supplementary Figure 5C). Compared with UPR, significant pathways identified in FPR4 mainly included nucleotide metabolism, tryptophan metabolism, glucosinolate biosynthesis, purine metabolism, phenylalanine metabolism, and pyruvate metabolism (Supplementary Figure 5D). Compared with FPR4, the FPR6 group showed enhanced metabolism in monobactam biosynthesis, cyanoamino acid metabolism, galactose metabolism, the biosynthesis of unsaturated fatty acids, arginine biosynthesis, and ABC transporters (Supplementary Figure 5E). Lastly, compared with FP6, the FP8 group mainly promoted metabolic processes such as the biosynthesis of unsaturated fatty acids, flavone and flavonol biosynthesis, phenylpropanoid biosynthesis, cyanoamino acid metabolism, tyrosine metabolism, and glutathione metabolism (Supplementary Figure 5F). Overall, glucosinolate biosynthesis, tryptophan metabolism, and nucleotide metabolism emerged as overlapping metabolic pathways identified through KEGG enrichment analysis across different comparison groups.

### 3.6 Analysis of volatile metabolites

#### 3.6.1 Basic qualitative classification of the volatile components in PW and PRW at different fermentation stages

HS–SPME/GC–MS/MS was used to investigate changes in the volatile composition of different fermented samples. The analysis identified 647 volatile compounds in both PW (UP, FP4, FP6, and FP8) and PRW (UPR, FPR4, FPR6, and FPR8) (Supplementary Table S10). These compounds included 130 terpenoids, 115 esters, 94 heterocyclic compounds, 55 ketones, 51 alcohols, 49 hydrocarbons, 44 aldehydes, 31 aromatics, 22 amines, 19 acids, 15 phenols, 6 nitrogen compounds, 6 sulfur compounds, 4 halogenated hydrocarbons, 4 ethers, and 2 other flavor substances. Notably, 1-tetradecanol, 2H-pyran, 3,6-dihydro-4-methyl-2-(2-methyl-1-propenyl)-, 5-amino-2-methoxyphenol, cyclohexene, and 3,4-diethenyl-1,6-dimethyl- were detected only in PRW. Quantitative GC–MS analysis results of the aroma substances are presented in Supplementary Table S11 and Table 1. The relative contents of volatile components in the fermented samples were significantly higher than those in the unfermented samples, indicating that yeast activity during fermentation increased the level of flavor substances, thereby enriching the flavor profile of fruit wines. In PRW, all volatile compounds showed an upward trend compared to PW, except for aromatic and sulfur compounds, which decreased. This difference in sensory quality, due to the interaction and combination of aroma and taste substances from pears and roses, can be attributed to the unique volatile compounds present in each, as well as their synergistic effects when combined. Studies have shown that the development of its characteristic quality and flavor profile is mainly fermentation-driven, with the fermentation process mediated by diverse microbial communities (47). The microbial communities involved in fermentation secrete diverse enzymatic systems that mediate oxidation, degradation, and polymerization reactions, playing a pivotal role in developing the distinctive aroma compounds characteristic of rose–pear wine (48).

**Table 1:** Types and relative contents of volatile compounds in different samples.

**Aroma component type**	**UP**	**FP4**	**FP6**	**FP8**	**UPR**	**FPR4**	**FPR6**	**FPR8**
**Contents (ug/L)**								
Ester	3,101.54	19,622.56	19,638.06	15,106.04	3,333.52	22,286.28	27,326.66	21,743.24
Amine	56.57	5,803.36	6,018.82	6,026.97	62.18	5,939.33	7,353.53	6,386.03
Alcohol	2,838.56	6,378.76	6,434.46	6,092.15	3,081.22	6,458.07	7,220.40	6,494.99
Aromatics	1,138.53	1,708.28	2,061.81	2,061.49	1,197.34	1,466.50	1,554.84	1,650.25
Phenol	861.51	899.09	952.20	927.41	1,041.21	1,028.61	1,025.23	1,010.22
Nitrogen compounds	145.48	195.39	193.52	187.29	218.22	350.44	387.50	385.54
Sulfur compounds	290.03	363.90	340.02	332.87	286.35	310.61	300.39	289.76
Halogenated hydrocarbons	353.73	564.34	589.81	627.21	284.74	584.27	702.89	634.86
Ether	29.24	125.39	131.54	117.03	93.15	143.49	159.24	136.77
Aldehyde	2,451.32	2,854.17	2,651.78	2,463.81	2,419.10	2,981.73	2,953.72	2,628.10
Acid	250.56	596.31	600.94	563.11	280.75	627.12	713.22	557.39
Terpenoids	5,267.24	6,852.35	7,157.39	6,238.13	6,293.43	8,010.04	8,537.02	7,669.62
Hydrocarbons	1,295.75	10,947.33	11,689.92	12,167.09	1,457.07	12,318.51	14,521.03	13,350.30
Ketone	1,049.18	5,314.83	5,675.58	5,276.82	1,153.11	5,708.99	6,817.29	5,910.36
Heterocyclic compound	5,762.30	6,144.19	6,174.45	5,915.27	5,980.98	6,648.31	6,680.23	6,350.04
Others	18.00	61.42	73.66	55.23	34.18	66.00	89.85	71.69

To explore the differences in volatile metabolites across fermented samples, a combination of univariate statistical analysis (FC), multivariate statistical analysis (PCA), and relative odor activity value (ROAV) assessment methods was applied. The PCA and HCA of the volatile flavor substances showed a distinct separation of samples in both PW and PRW, consistent with the findings for non-volatile flavor substances (Supplementary Figures 6A, B).

#### 3.6.2 Evolution of different metabolites during fermentation

**Figure 5:**
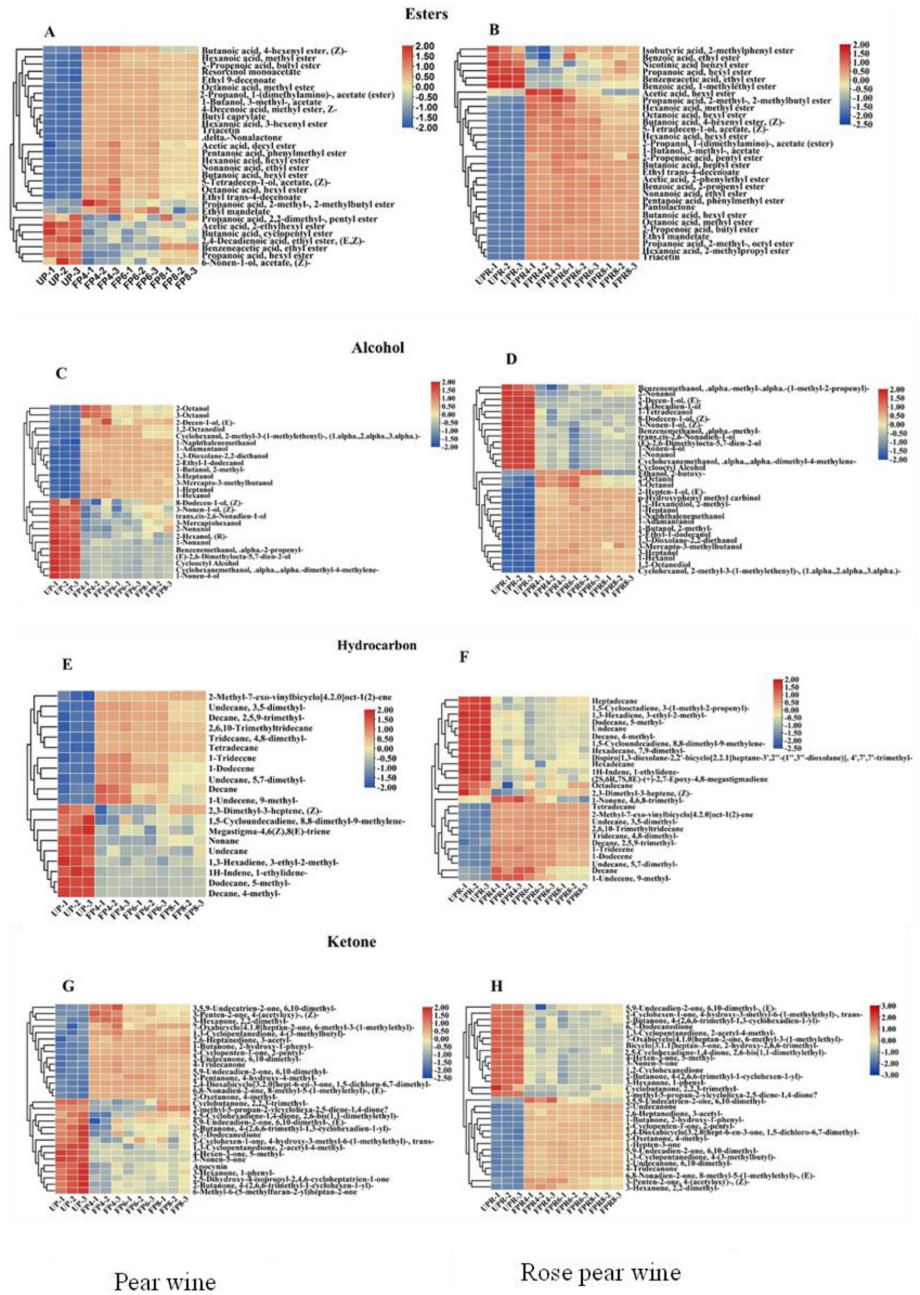
Heatmaps of the contents of significantly different esters **(A, B)**, alcohol **(C, D)**, hydrocarbons **(E, F)**, and ketones **(G, H)** in PW and PRW during fermentation. Each colored cell corresponds to a value of different categories of non-volatile metabolites. Red color indicates high content, while green color indicates low content.

Esters are the primary contributors to the aroma quality during fermentation, imparting the fruity and floral characteristics typical of fruit wines (49). They are formed through the esterification of acids and alcohols or by the action of alcohol acetyltransferase on substrates containing higher alcohols and acetyl-CoA (50). As shown in Figures 5A, B, the concentration of esters in PW and PRW initially increased with fermentation time, followed by a subsequent decrease. Decanoic acid ethyl ester had the highest content among the esters, reaching 7,578.83 ± 656.7 μg/L in FP4 and 11,202.08 ± 1,049.03 μg/L in FPR6. Esters showed the highest concentration and the most significant changes among all aroma compounds detected. Compared with UP, the FP4 group had increased levels of esters like ethyl 9-decenoate, which imparts a fruity aroma, and resorcinol monoacetate. As fermentation progressed, additional esters, such as 2-propenoic acid and 2-methoxyethyl ester, were detected in FP6. Compared with UPR, the FPR4 group contained higher levels of decanoic acid ethyl ester, which imparts sweet, waxy, fruity, apple, grape, oily, and brandy aromas. As fermentation continued, compounds such as butanoic acid 3-hexenyl ester (Z), which imparts fresh, green, apple, fruity, wine, metallic, and buttery aromas, and butanoic acid 3-hexenyl ester (E) were detected in FPR6. However, the levels of certain esters, including propanoic acid hexyl ester, 1-ethylpropyl acetate, 2-hexen-1-ol acetate (E), 3-mercaptohexyl acetate, and acetic acid cyclohexyl ester, decreased. This reduction may be due to these esters undergoing complex reactions as precursors for the production of other volatile flavor substances. Additionally, butanoic acid 3-hexenyl ester (E) and butanoic acid 3-hexenyl ester (Z) were not detected at the 4-day fermentation stage but appeared at the 6-day stage, indicating their formation through yeast metabolism during the later stages of fermentation (51).

Alcohols are fundamental aroma compounds that significantly contribute to the complexity of fruit wine. These substances are key components in fermented wines and are synthesized through pathways such as the glycolytic pathway, amino acid metabolism (the Ehrlich pathway), methyl ketone reduction, and the degradation of linoleic acid and linolenic acid (52). As shown in Figures 5C, D, alcohol levels in PW and PRW initially increased with fermentation time before subsequently decreasing. The concentration of 1-butanol 2-methyl reached a maximum value of 2,687.79 ± 61.57 μg/L in FP8 and 2,963.79 ± 197.51 μg/L in FPR6. This compound, which imparts winey, whiskey, and cocoa characteristics, significantly contributed to the sensory complexity of both PW and PRW. In this study, alcohols such as 1,2-octanediol, 1,3-dioxolane-2,2-diethanol, 1-butanol 2-methyl-, 1-heptanol, 1-hexanol, 1-naphthalenemethanol, 2-hepten-1-ol (E), and 3-heptanol were present in FP4 and FPR4, with their concentrations significantly increasing during fermentation, indicating that they were primarily derived from the fermentation process.

Hydrocarbons, particularly unsaturated olefins, decompose into alcohols, aldehydes, and keto acids during the brewing process, significantly contributing to the quality of fruit wine (53). As shown in Figures 5E, F, 2-methyl-7-exo-vinylbicyclo [4.2.0]oct-1(2)-ene significantly increased after fermentation, reaching a maximum value of 10,185.97 ± 436.89 μg/L in FP8 and 11,266.37 ± 327.71 μg/L in FPR6. Tetradecane (mild, waxy aroma) was detected in FP4 and FPR4, while cubenene (spicy, fruity, mango aroma) was detected in PW. The overall variety and concentration of hydrocarbon flavor substances were relatively small, likely due to their oxidation into other flavor substances during fermentation. This observation highlights the role of hydrocarbons in the brewing process of PW and PRW.

Ketones, which have strong odors, are mainly formed from the β-oxidation of saturated fatty acids or the degradation of amino acids during fermentation (54). As shown in Figures 5G, H, 2-oxetanone 4-methyl- significantly increased after fermentation, reaching a maximum value of 3,184.87 ± 118.12 μg/L in FP6 and 3,835.06 ± 172.76 μg/L. Over time, the compound 3,5,9-undecatrien-2-ol, 1,6,10-trimethyl- in PW develops a sweet, waxy, citrusy, floral, and spicy aroma.

#### 3.6.3 ROAV

The ROAV is a method used to identify key flavor compounds in food by considering the sensory thresholds of these compounds to determine their contribution to the overall aroma characteristics of a sample. To further evaluate the odor intensity of each volatile compound, we calculated the ROAV for both PW and PRW. In general, ROAV ≥ 1 indicates that a compound makes a direct contribution to the flavor of the sample, whereas volatile components with ROAV values between 0.1 and 1 may not directly contribute to aroma formation but can still enhance the overall flavor profile of the sample (Supplementary Table S12). The analysis showed that 73 volatile compounds in PW and PRW had concentrations above their respective sensory thresholds. Key aroma components included compounds such as 2(5H)-furanone, 5-ethyl-3-hydroxy-4-methyl-, 2-thiophenemethanethiol, 2-nonenal, decanoic acid ethyl ester, pyrazine, 2-methoxy-3-(2-methylpropyl)-, dodecanenitrile, furaneol, 2-buten-1-one, 1-(2,6,6-trimethyl-1,3-cyclohexadien-1-yl)-, (E)-, 2,6-nonadienal (E,Z)-, 1-hepten-3-one, 1-butanol, 2-methyl-, trans, cis-2,6-nonadien-1-ol, 1-butanol, 3-methyl-, acetate, and 2-nonenal. These compounds had ROAV values higher than 100, suggesting their significant role as primary contributors to the aroma profile of PPW and PRW. Moreover, the ROAV values for PRW were generally higher than for PW, indicating a stronger odor contribution. Decanoic acid ethyl ester, characterized by sweet, waxy, fruity, apple, grape, oily, and brandy-like aromas, was a distinctive flavor component of both PW and PRW, significantly enhancing the overall sensory complexity of the samples (20, 55).

### 3.7 Sensory analysis

The sensory evaluation score of PRW was 87.9, and the corresponding sensory analysis results are shown in Supplementary Figures 7A, B. PW was characterized by a moderate intensity of most rated attributes, including waxy, floral, herbal, and woody notes, as well as a lower intensity of spicy, fresh, and tropical aromas. It also exhibited a strong perception of green, fruity, and sweet characteristics. Similarly, PRW displayed a moderate intensity for waxy, floral, herbal, and woody attributes but lower levels of fatty, fresh, and tropical notes, with a relatively strong perception of green, fruity, and sweet characteristics. Although the “fruity” and “sweet” aromas were rated relatively higher for both PW and PRW, the “spicy” and “green” descriptors also received higher scores, contributing to a decrease in the overall aroma intensity in PW. The sensory evaluation indicated that the overall aroma of PRW was significantly enhanced. This enhancement can be attributed to the release and perception of aroma compounds, which depend not only on the volatile compounds present but also on the non-volatile substances in the fruit wine, such as polyphenols, amino acids and their derivatives, lipids, and other components.

## 4 Conclusions

The sensory characteristics of fruit wine are primarily determined by taste and aroma, both closely related to a diverse range of flavor compounds. In conclusion, the experimental results indicated that the acidity and soluble solids content in the compound fruit wine were moderate. The total phenol and flavonoid contents, antioxidant capacities, and tyrosinase inhibition abilities significantly increased in compound wine. Compared to P, PL, PC, PD, and PO, the PR exhibited a greater effect. Furthermore, a total of 2,401 non-volatile metabolites, including 11 subclasses, and 647 volatile metabolites, including 16 subclasses, were identified in both PW and PRW, with PRW producing more volatile and non-volatile metabolite compounds than PW. Further analysis clarified the evolutionary trajectories of non-volatile metabolites, revealing that the rapid transformation of flavonoids and phenolic acids was a main determinant influencing the color, astringency, taste, flavor, and overall appearance of compound fruit wine. The differential metabolites shared among different comparison groups were primarily enriched in pathways related to tryptophan metabolism, glucosinolate biosynthesis, and nucleotide metabolism. These pathways play a vital role in affecting the characteristics of compound fruit wines prepared from different edible flowers and fruits. In addition to the observed changes in volatile metabolites, esters, hydrocarbons, alcohols, and ketones significantly contributed to the formation of aromatic substances, with decanoic acid ethyl ester making a significant contribution to the profile of the fruit wine. This enhancement contributed to the fruity, floral, aromatic, and brandy aromas of the wine. Sensory analysis also showed that PRW exhibited superior aroma propagation compared to PW. The findings of this research provide valuable insights for the development of polyphenol-based food products and serve as a reference for quality control and flavor research in compound fruit wine products. Further exploration of the genetic or metabolic engineering of fermentation microbes could enhance desirable pathways (e.g., tryptophan metabolism and glucosinolate biosynthesis) for improved aroma and bioactive compound production.

## Data Availability

The original contributions presented in the study are included in the article/supplementary material, further inquiries can be directed to the corresponding author.
