# Supplementary material for: Influence of edible flower inclusion on the nutritional and flavor changes of fermented pear wine
Source: Front Nutr. 2025 Jul 21;12:1604754. doi: 10.3389/fnut.2025.1604754 (PMC12318767; doi:10.3389/fnut.2025.1604754)
Supplement: Supplementary file 2 [file Image_1.pdf]

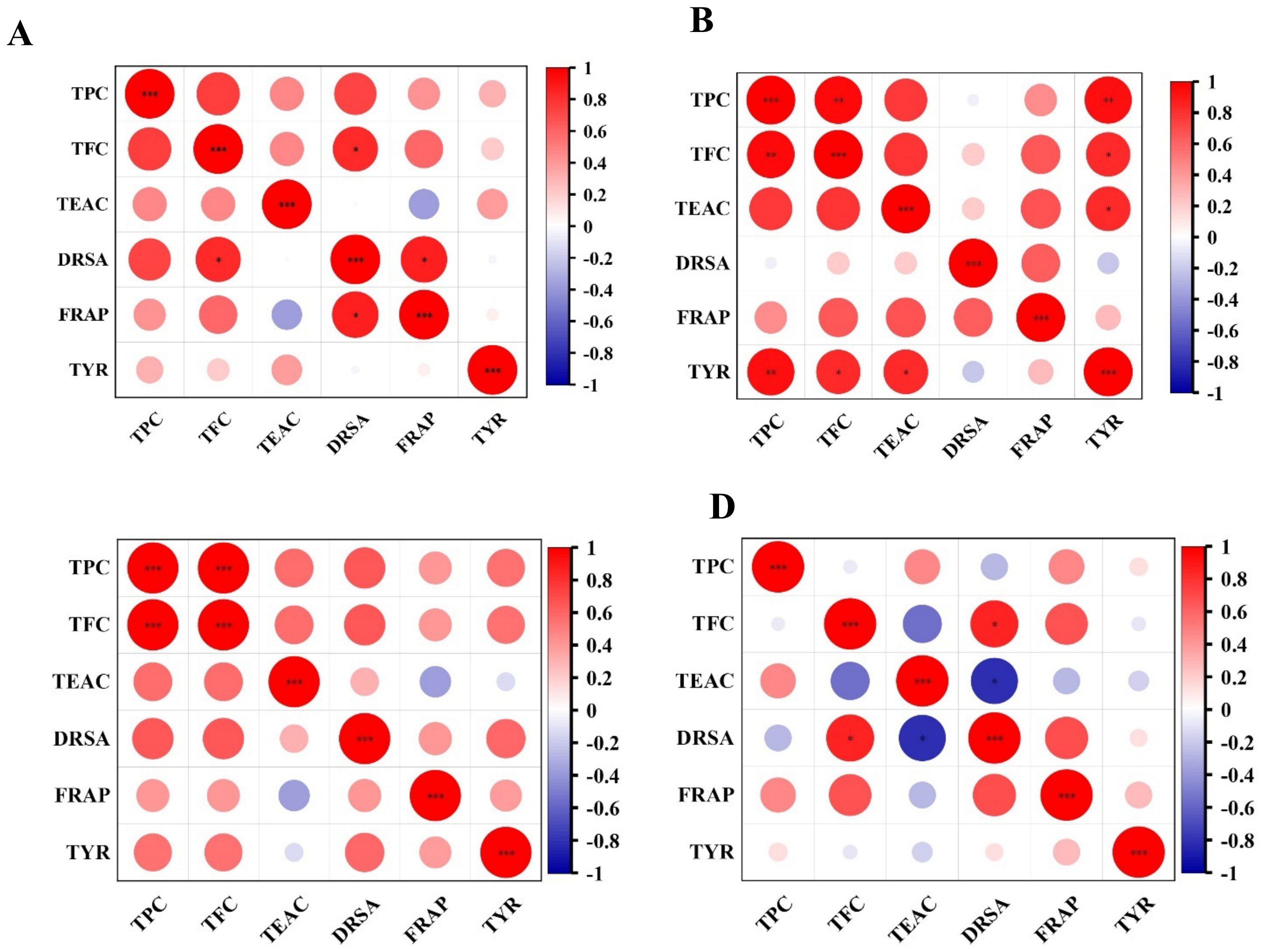

A
B
C
D
TPC
TFC
TEAC
DRSA
FRAP
TYR
1
0.8
0.6
0.4
0.2
0
-0.2
-0.4
-0.6
-0.8
-1

E F G

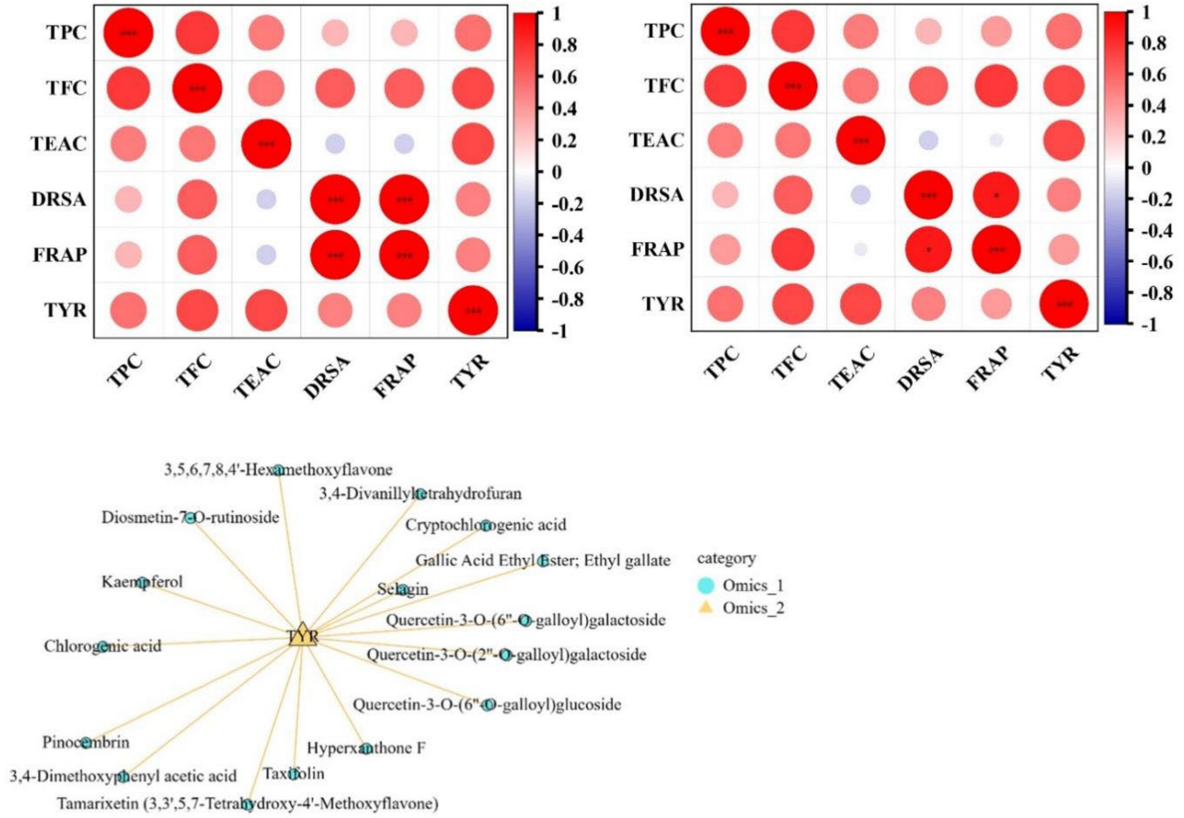

***** p<=0.05 ** p<=0.01 *** p<=0.001**

Supplementary Fig. 1 Correlation analysis between the change of TPC, TFC, antioxidant activity and tyrosinase inhibition activity during fermentation: A (P), B(PR), C(PD), D(PO), E(PL), F(PC) and G (Correlation network diagram of PR and TYR)

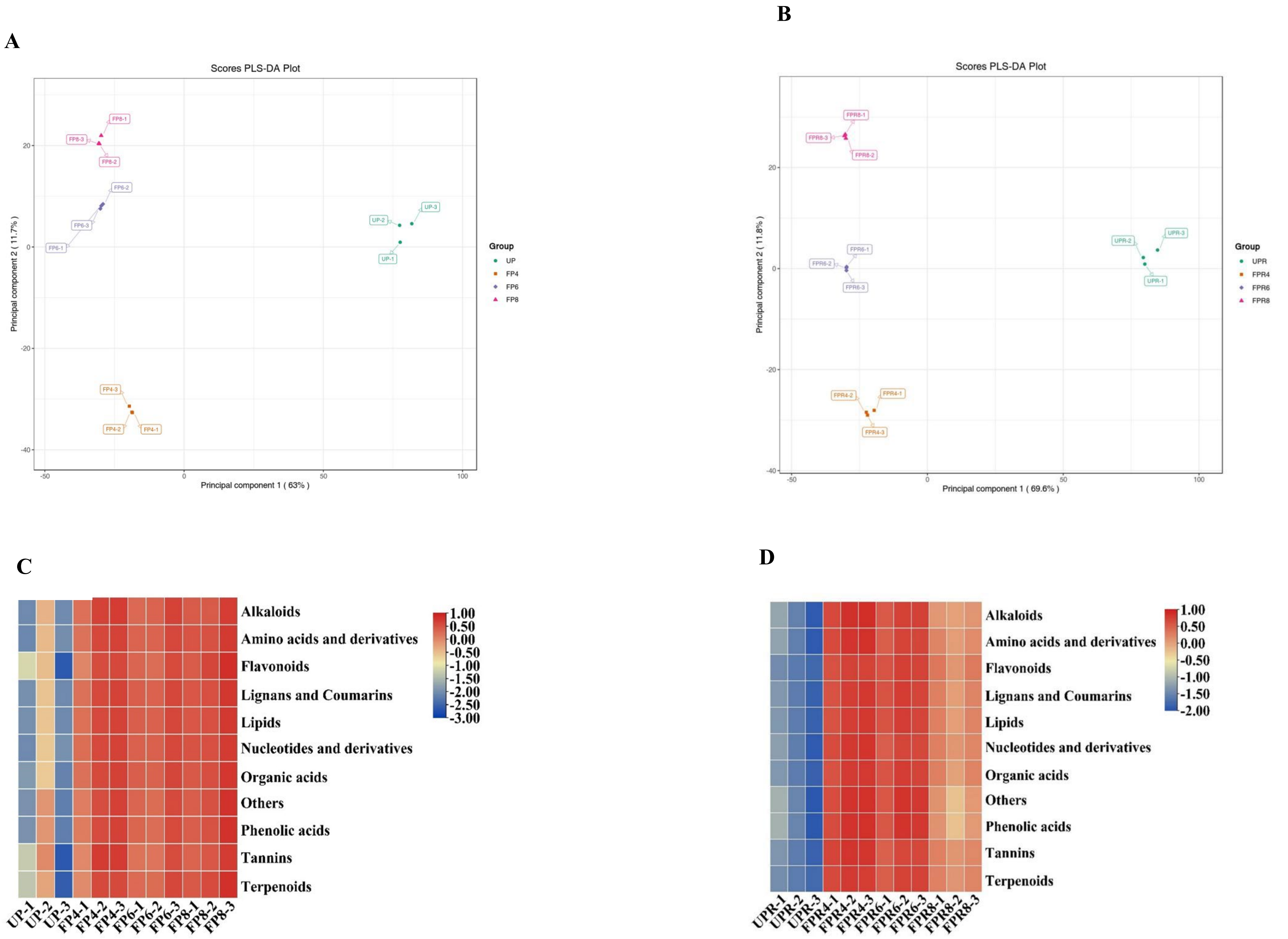
A
Scores PLS-DA Plot
Principal component 1 (63%)
Principal component 2 (11.7%)
Group
UP
FP4
FP6
FP8
B
Scores PLS-DA Plot
Principal component 1 (69.6%)
Principal component 2 (11.8%)
Group
UPR
FPR4
FPR6
FPR8
C
Alkaloids
Amino acids and derivatives
Flavonoids
Lignans and Coumarins
Lipids
Nucleotides and derivatives
Organic acids
Others
Phenolic acids
Tannins
Terpenoids
UP-1 UP-2 UP-3 FP4-1 FP4-2 FP4-3 FP6-1 FP6-2 FP6-3 FP8-1 FP8-2 FP8-3
1.00 0.50 0.00 -0.50 -1.00 -1.50 -2.00 -2.50 -3.00
D
Alkaloids
Amino acids and derivatives
Flavonoids
Lignans and Coumarins
Lipids
Nucleotides and derivatives
Organic acids
Others
Phenolic acids
Tannins
Terpenoids
UPR-1 UPR-2 UPR-3 FPR4-1 FPR4-2 FPR4-3 FPR6-1 FPR6-2 FPR6-3 FPR8-1 FPR8-2 FPR8-3
1.00 0.50 0.00 -0.50 -1.00 -1.50 -2.00

Fig.2. Partial least squares discrimination analysis (PLS-DA): pear wine(A), rose pear wine (B). Heatmap of the changes in non-volatile metabolites in pear wine(C) and rose pear wine(D) during fermentation. Each colored cell corresponds to a value of different categories of non-volatile metabolites. Red color indicates high content, while green color indicates low content.

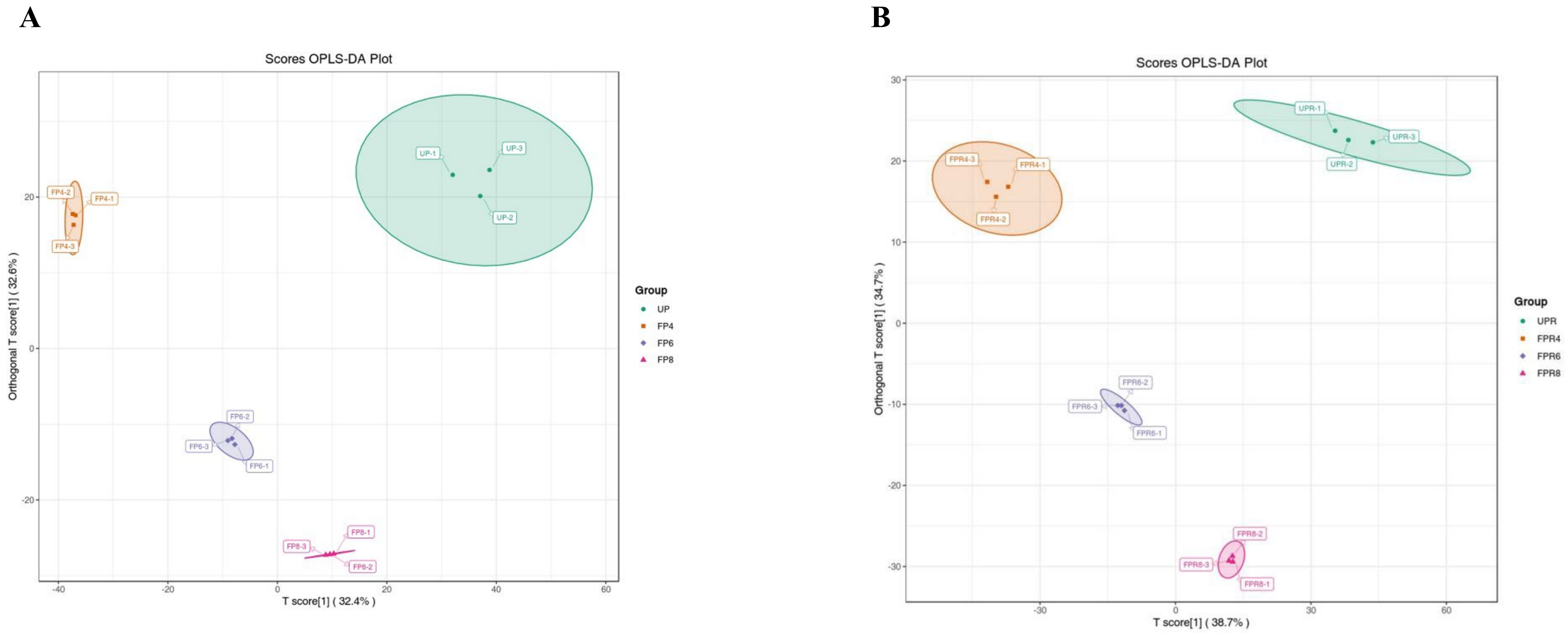

Supplementary Fig3. OPLS-DA score plots of samples for different fermentation periods: pear wine(A), rose pear wine(B)

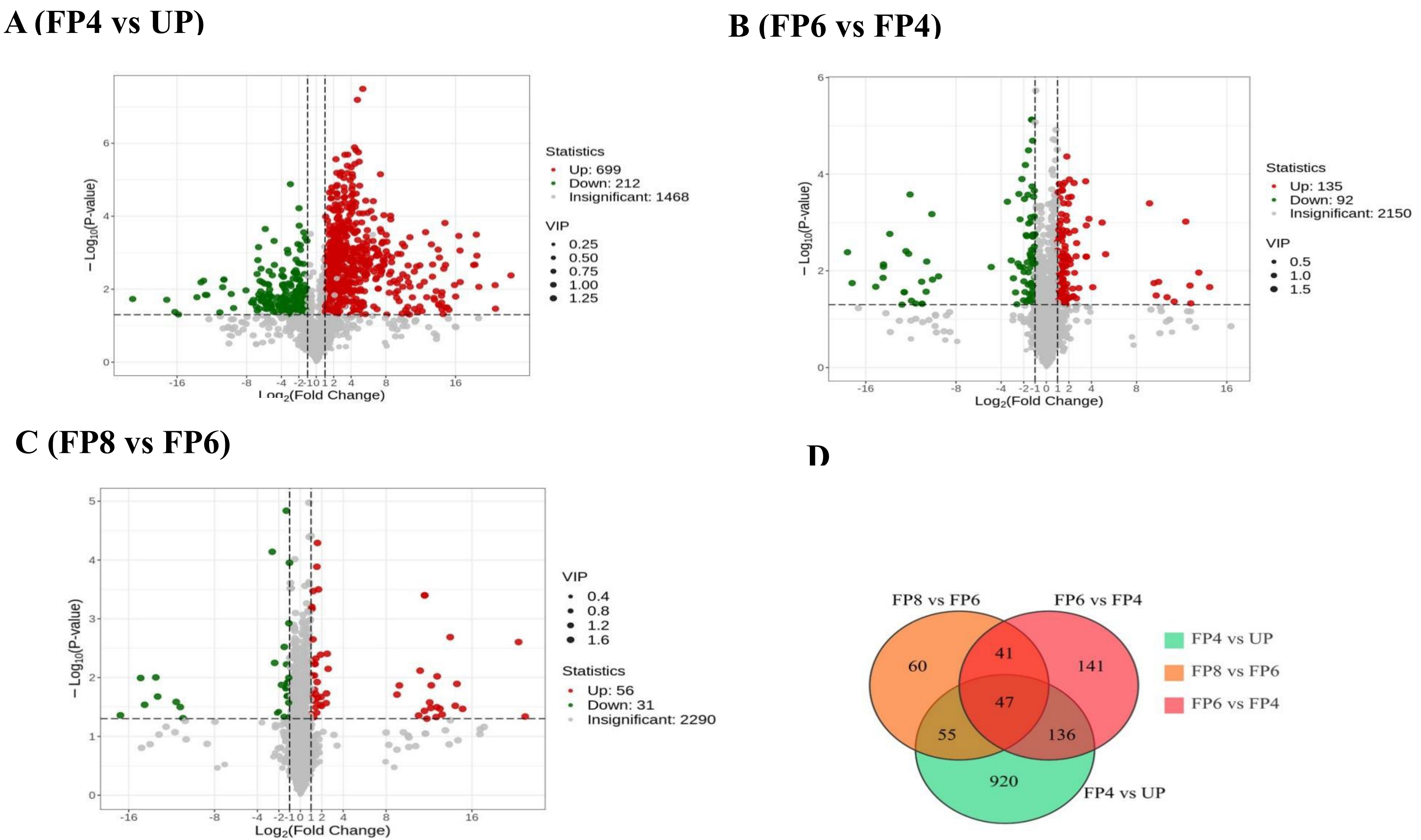

Supplementary Fig. 4-1 Volcano plot (A, B, C,) and Venn diagram (D) in pear wine. The fire mountain map with the red dots represent significantly up-regulated differential metabolites, blue dots represent significantly down-regulated differential metabolites, and grey dots represent insignificant differential metabolites. The numbers in the Wayne diagram indicate the number of intersecting biological metabolites.

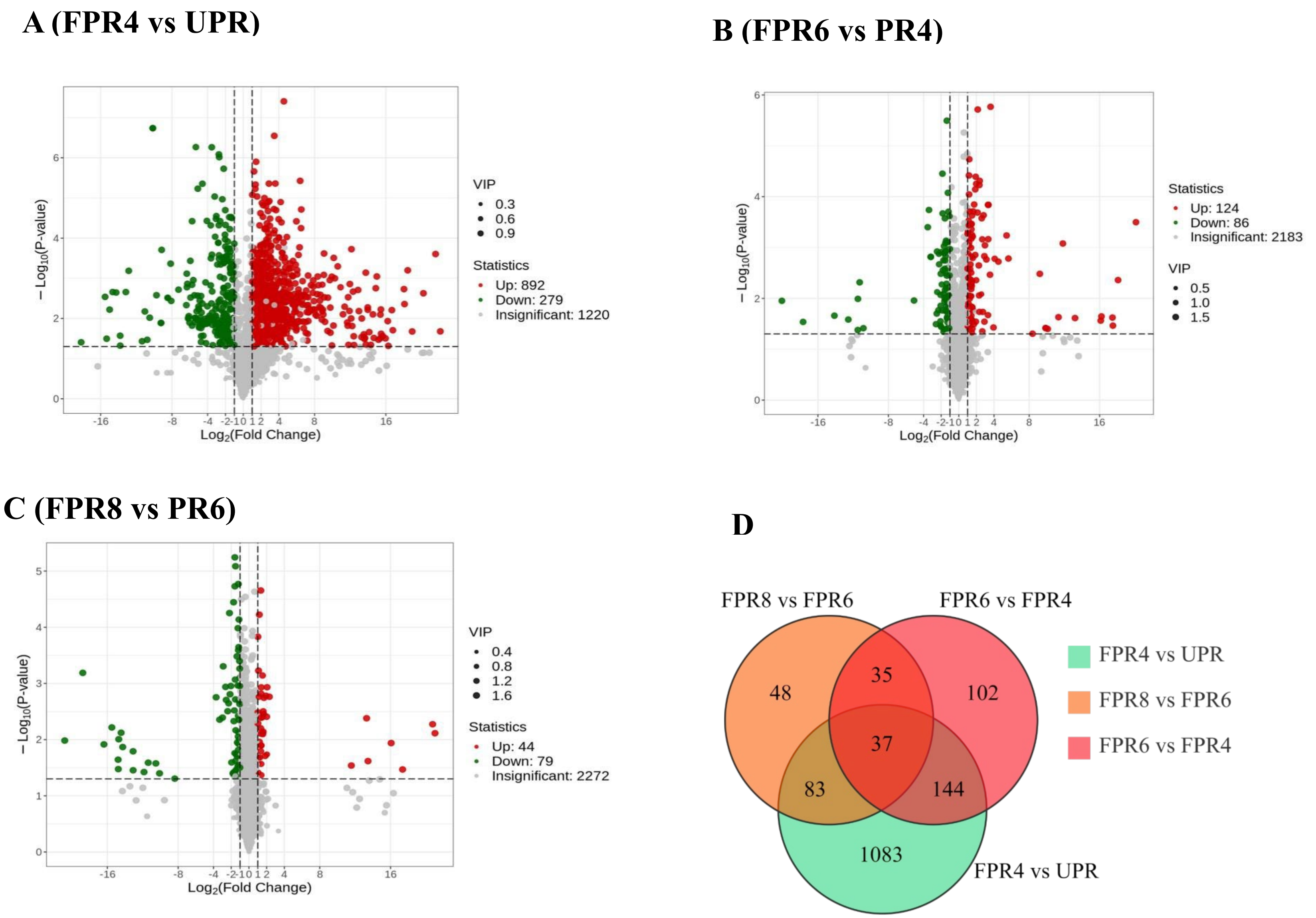

Supplementary Fig.4-2 **Volcano plot (A, B, C,) and Venn diagram (D) in rose pear wine. The fire mountain map with the red dots represent significantly up-regulated differential metabolites, blue dots represent significantly down-regulated differential metabolites,**

and grey dots represent insignificant differential metabolites. The numbers in the Wayne diagram indicate the number of intersecting biological metabolites.

A (FP4 vs UP)

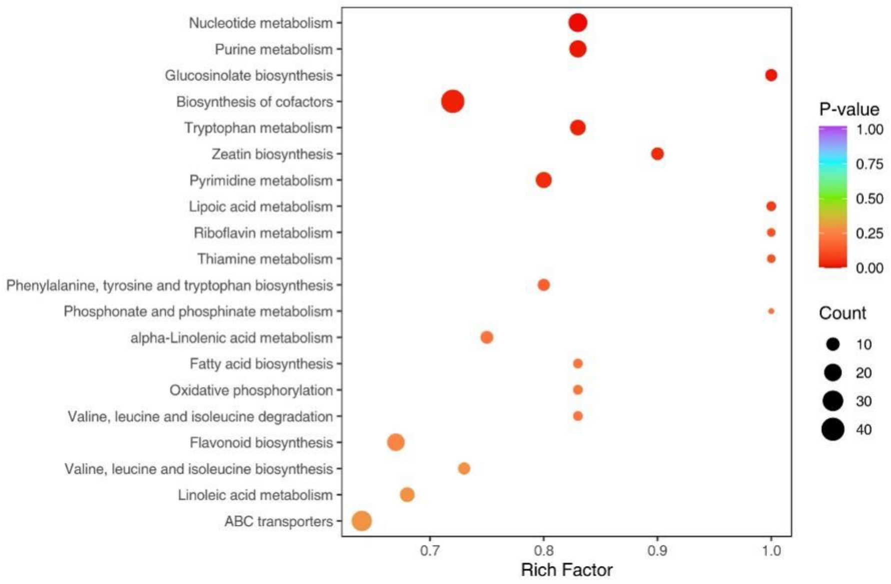

B (FP6 vs FP4)

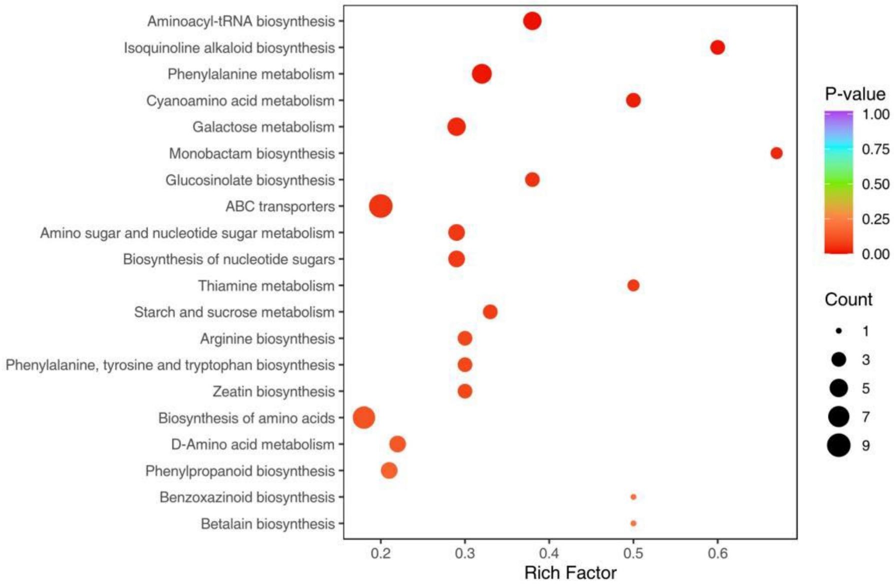

C (FP8 vs FP6)

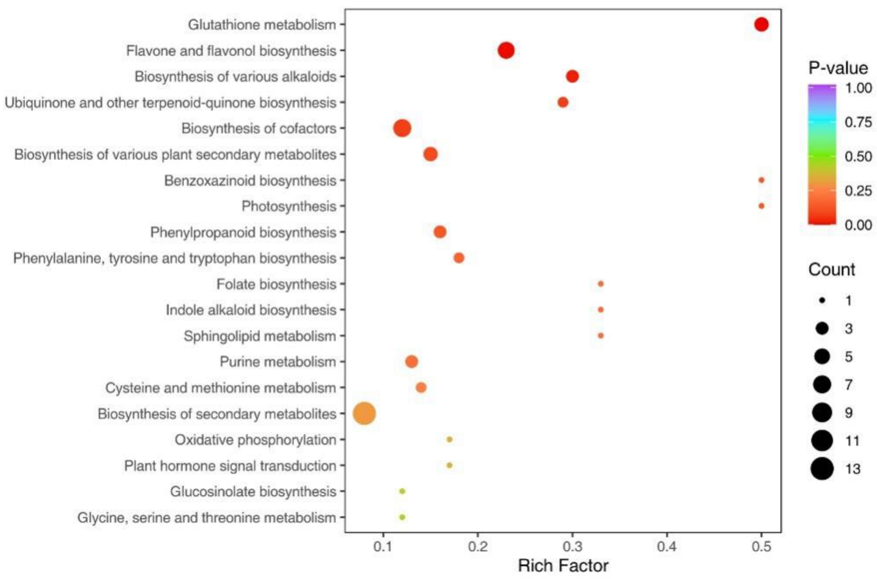

D (FPR4 vs UPR)

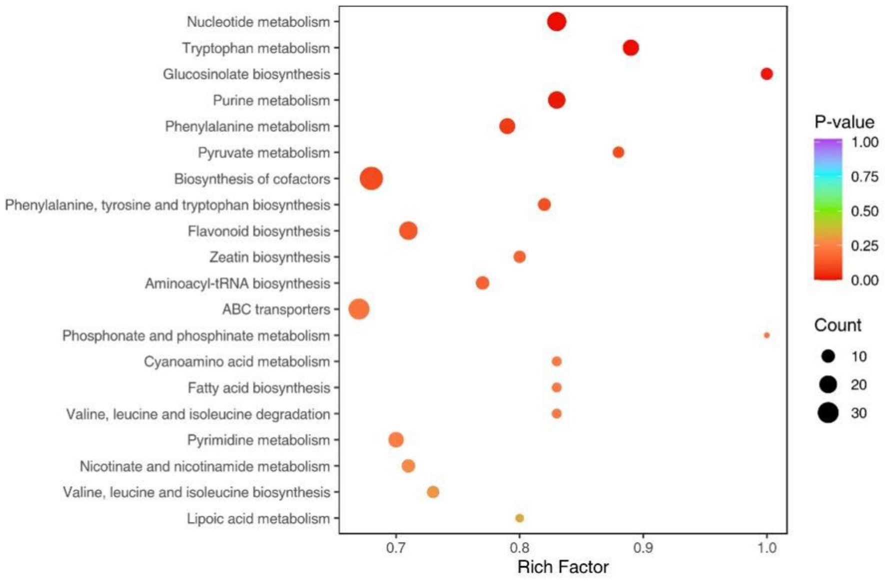

D (FPR6 vs PR4)

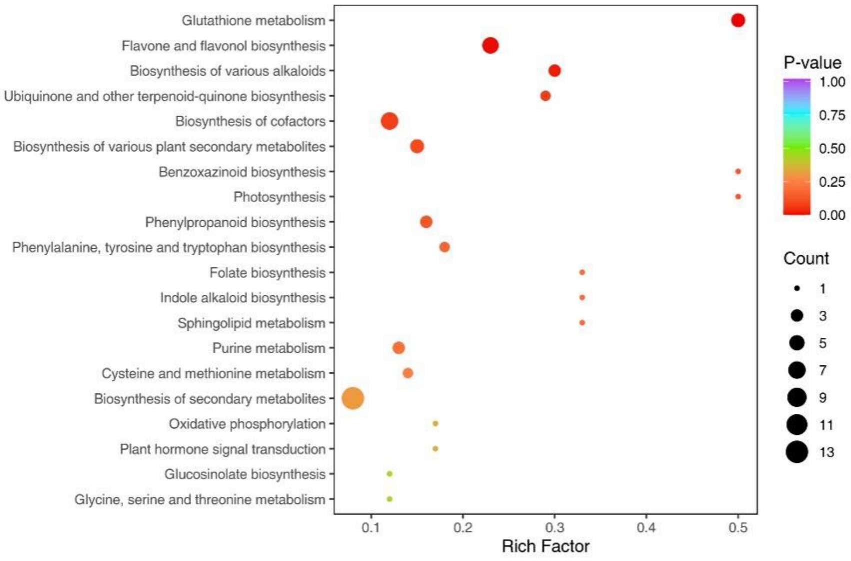

D (FPR8 vs PR6)

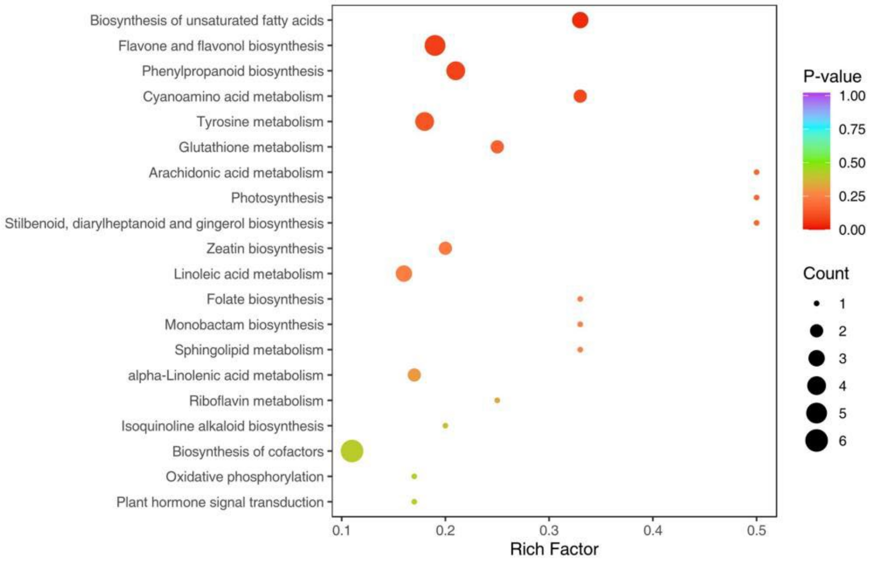

Supplementary Fig. 5 **KEGG pathway annotation of the differential metabolites: pear wine (A-C), rose pear wine(D-F)**

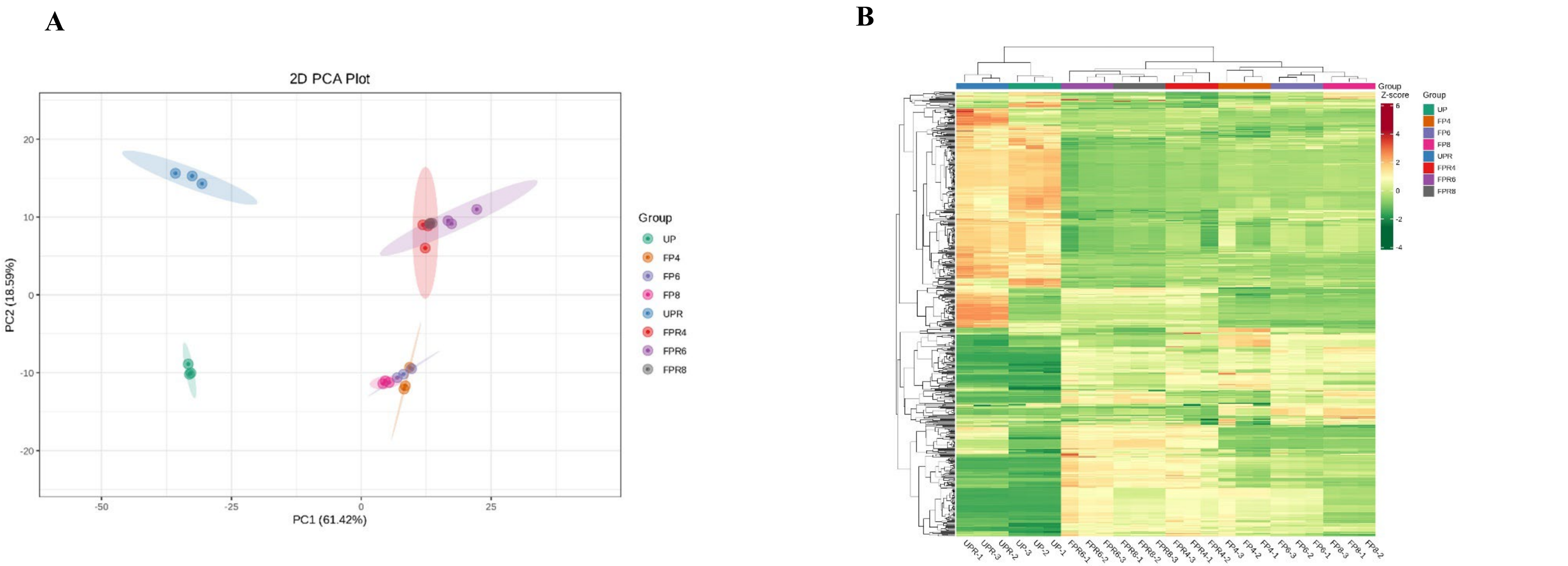

Fig.6 PCA score plot of all samples (A) and Heatmap of the changes in non-volatile metabolites in pear wine and rose pear wine during fermentation.

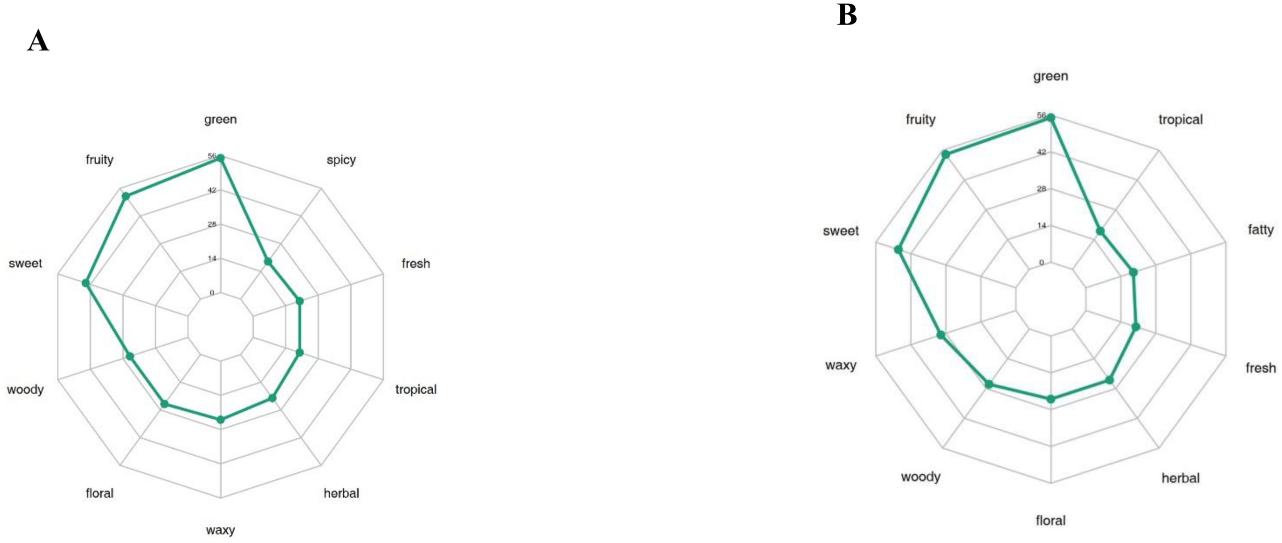

Fig.7. Average values of sensory evaluation scores of PW(A) and PRW(B).
